# In silico prediction of the interaction of legacy and novel per- and poly-fluoroalkyl substances (PFAS) with selected human transporters and of their possible accumulation in the human body

**DOI:** 10.1007/s00204-024-03797-0

**Published:** 2024-06-17

**Authors:** G. A. Tiburtini, L. Bertarini, M. Bersani, T. A. Dragani, B. Rolando, A. Binello, A. Barge, F. Spyrakis

**Affiliations:** 1 Department of Drug Science and Technology, University of Turin, Turin, Italy; 2 Present Address: Department of Life Sciences, University of Modena and Reggio Emilia, Modena, Italy; 3 Aspidia Srl, 20100 Milan, Italy

**Keywords:** Molecular docking, Molecular dynamics, HSA, OAT, PFAS, PFOA, PFOS, cC6O4

## Abstract

Per- and poly-fluorinated compounds constitute a wide group of fluorocarbon chemicals with widespread industrial applications, ranging from non-stick coating in cookware to water surfactants, from fire-fighting foams to water-repellent coatings on textiles. Presently, over 12,000 PFAS are known worldwide. In recent years, extensive research has focused on investigating the biological effects of these molecules on various organisms, including humans. Here, we conducted in silico simulations to examine the potential binding of a representative selection of PFAS to various human proteins known to be involved in chemical transportation and accumulation processes. Specifically, we targeted human serum albumin (HSA), transthyretin (TTR), thyroxine binding protein (TBG), fatty acid binding proteins (FABPs), organic anion transporters (OATs), aiming to assess the potential for bioaccumulation. Molecular docking simulations were employed for this purpose, supplemented by molecular dynamics (MD) simulations to account for protein flexibility, when necessary. Our findings indicate that so-called “legacy PFAS” such as PFOA or PFOS exhibit a higher propensity for interaction with the analysed human protein targets compared to newly formulated PFAS, characterised by higher branching and hydrophilicity, and possibly a higher accumulation in the human body.

**Supplementary Information:**

The online version contains supplementary material available at 10.1007/s00204-024-03797-0.

## Introduction

According to the *Organisation for Economic Co-operation and Development* (OECD), per- and poly-fluorinated alkyl substances (PFAS) are chemicals that contain at least one fully fluorinated methyl (–CF3) or methylene (–CF2–) carbon atom (without any H/Cl/Br/I atom attached to it), with a few noted exceptions (OECD 2021). Well-known for their numerous industrial applications, PFAS show poor affinity with both polar and apolar substances, high chemical and thermal resistance, and enough chemical and physical stability to likely become long-lasting chemicals (Vecitis et al. 2009; Göckener et al. 2020; Meneguzzi et al. 2021; Leung et al. 2023). Of particular interest is their protein-philic character (Gao et al. 2019; Sharma et al. 2022), which arises from the hydrophobicity of their per/poly-fluorinated chain, the hydrophilicity of their functional group and from the peculiar interaction between a per-fluorinated methyl group, i.e. the last carbon of their chain, and a carbonyl group in proteins’ backbone. This distinctive feature has generated concerns on PFAS possible accumulation in living tissue. Several studies investigated the interaction of legacy PFAS, i.e. perfluorooctanoic acid (PFOA) and perfluorooctane sulfonic acid (PFOS), with specific proteins as human serum albumin (HSA), fatty acid binding proteins (FABPs), organic anion transporters (OATs), by means of experimental methodologies (Zhang et al. 2013a; Sheng et al. 2016; Gao et al*.* 2019; Xu et al. 2020; Fenton et al. 2021; He et al. 2023). These analyses, even if useful, are all but comprehensive, usually encompassing a handful of substances, and the available data seem insufficient to obtain a conclusive understanding of the potential bioaccumulation of different PFAS categories.

To provide a more general view of PFAS possible accumulation in the body, we applied an in silico approach to study the interaction of possible protein targets with a representative set of legacy and newly formulated PFAS.

### PFAS categories

Our dataset (see Table 1 for common names and Table S1 for IUPAC names) was chosen to properly coverage different PFAS categories. It includes carboxylic and sulfonic acids, which are the most studied fluorocarbon substances, but also poly-fluorinated alcohols (fluorotelomers), per-fluorinated alkanes with no functional groups and per- and poly-fluorinated alkyl ethers. The latter group was included because it encompasses the majority of novel PFAS, in particular GenX and cC6O4. Classification of PFAS into different groups, as attained in this work, helps differentiate these compounds according to physical–chemical properties, and contribute to correctly assessing their potential risks and health hazards (Cousins et al. 2020). Accordingly, many governmental dossiers used similar groupings to evaluate group-level hazards (OECD 2021; UK REACH 2023). For the sake of simplification, we also subdivided our molecules in two macro categories, which have been extensively used in scientific literature (Sheng et al. 2018; Perera et al. 2022; He et al. 2023). The so-called “legacy PFAS” are per-fluorinated alkyl chains, as PFOA and PFOS, varying in length with diverse functional groups, extensively studied in the past decades for their potential bioaccumulation. “Newly formulated” or “novel” PFAS, on the other hand, are shorter, branched and bulkier molecules with a carboxylic acid moiety, developed as an alternative to linear molecules.

**Table 1:**
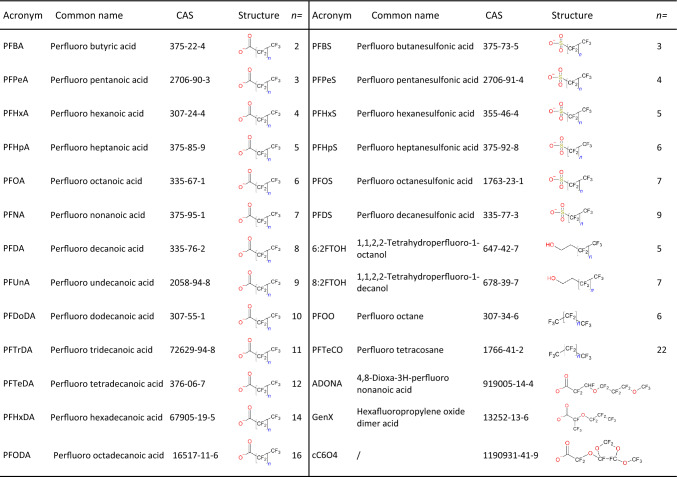
List of analysed PFAS

### Target proteins

Proteins known to be involved in chemical transportation and accumulation have been included in the study: HSA, transthyretin (TTR), thyroxine binding protein (TBG), FABPs and OATs. Their role and importance are briefly described hereafter.

Human serum albumin (HSA) is the most abundant protein in the human serum, accounting for 60% of all serum proteins and weighting a total mass of 35–55 g per litre of blood. The main HSA functions are colloidal osmotic pressure regulation and transportation of a variety of poorly soluble compounds (Kragh-Hansen et al. 2002). Importantly, HSA is known to be PFAS primary vector in the human body (Forsthuber et al. 2020; Crisalli et al. 2023), thanks to PFAS similarity to fatty acids (Gao et al*.* 2019; Fenton et al. 2021; Alesio et al. 2022).

Transthyretin (TTR) is a highly conserved, homo-tetramer protein found in human serum and cerebrospinal fluid. Its functions, which define its name, are thyroxine (T4) and retinol binding protein (RBG) transportation (Refetoff 2000).

Thyroxine binding globulin (TBG) is the least abundant of the thyroxine binding proteins but has the highest affinity for both T4 and its de-iodinated form T3 (Refetoff 2000). It has a single T4 and T3 binding pocket on its surface, and it is responsible for transporting the majority of T4 in the blood, despite its low concentration.

Fatty acid binding proteins (FABPs) belong to a family of ten small proteins found in different tissue cells’ cytosol and are fundamental for the transport of fatty acids and other lowly soluble substances. Despite their name, in fact, FABPs can transport a variety of ligands as, for instance, retinoids and bile acids (Refetoff 2000). Five FABPs, being the most expressed and studied proteins of this family, have been included in the study: liver FABP (L-FABP), intestinal FABP (I-FABP), heart FABP (H-FABP), adipocyte (A-FABP) and peripheral myelin protein 2 (PmP2). They can also be expressed on different tissues other than the one giving them their name; for example, L-FABP is extensively expressed in the intestine as well, whilst H-FABP is expressed throughout all skeletal muscle tissue (Smathers and Petersen 2011).

Organic anion transporters (OATs) belong to the SLC22A superfamily, which comprises OAT1, OAT3, OAT4 and uric acid transporter 1 (URAT1), amongst others (Li et al. 2019). OATs are transmembrane proteins formed by 12 helices, found primarily in the kidney, placenta and brain; their primary function is to transport negatively charged molecules through the membrane walls. In particular, they are antiporters and mediate the transport of uric acid and steroids like estrone sulphate (Li et al. 2019; Zhao et al. 2020; Janaszkiewicz et al. 2023). They vary for molecular weight, sequence length and, in the kidney, for their function as well. OAT1 and OAT3 are located on the tubule basolateral membrane and transport solutes from the blood to the cytosol for subsequent urine excretion. OAT4 and URAT1 are located on the tubule apical membrane and are involved in solute transport (reuptake) from the urine to the bloodstream.

## Materials and methods

### Protein structures

Crystallographic protein structures were retrieved, when available, from the Protein Data Bank (PDB) (Berman et al. 2000): HSA, PDB ID 7AAI; TTR, PDB ID 5JID; TBG, PDB ID 4X30; L-FABP, PDB ID 3STK; I-FABP, PDB ID 2MO5; H-FABP, PDB ID 4TKJ; A-FABP, PDB ID 3P6D; PmP2, PDB ID 6S2S. The structural models of OAT1, OAT3 and OAT4, given the absence of experimental information, were obtained by homology modelling using i-TASSER bioinformatics server (Yang and Zhang 2015; Zheng et al. 2021). The structure of URAT1 was kindly provided by Zhao et al. (Zhao et al. 2020). Proteins were prepared with the Protein Preparation Wizard workflow in Maestro 2022-4 (Madhavi Sastry et al. 2013). Protonation states of charged residues, H-bond network and water molecule orientation were fixed.

### PFAS structures

Liu et al. (2021) reported a list of 18 linear PFAS they used to study drinking water contamination, including both carboxylic and sulfonic acids (Liu et al. 2021). To these, we added two fluorotelomeric acids (6:2FTOH and 8:2FTOH), two perfluoroalkanes with no functional group (named by us PFOO and PFTeCO) and three newly formulated, branched PFAS (commercially named ADoNA, GenX and cC6O4). The list of studied PFAS, their acronym, common/commercial name and IUPAC name are reported in Table 1 and Table S1, respectively. The 3D structures of investigated PFAS were retrieved from PubChem (Bolton et al. 2011) when available; otherwise, their 2D structures were drawn using the MolDraw software (Ugliengo 2006); Maestro 2022-4 package was used for 3D modelling and chirality specification. In the presence of chiral centres, all stereoisomers were modelled. Per-fluorinated molecules exhibit unique chemical properties dissimilar from any hydrogenated alkyl molecule of equal length as, in particular, the gauche effect (Cormanich et al. 2017; Leung et al. 2023), their carbon chain rigidity, far superior to that of corresponding fatty acids and the atomic charges disposition. These properties are not correctly represented by conventional molecular mechanics force fields (Borodin et al. 2002). We, thus, calculated PFAS geometries and atomic partial charges applying a DFT level of complexity, using the Jaguar software package (Bochevarov et al. 2013), with B3LYP functional and the Pople’s basis set 6–31++G**.

### Molecular docking

All ligands reported in Table 1 were submitted to rigid docking (RD) (Friesner et al. 2004, 2006) and induced fit docking simulations (IFD) (Sherman et al. 2006a, b) in all mentioned proteins, using the Schrodinger suite, version 2022-1. The binding site was chosen according to crystallographic and literature information about native ligand binding mode, when available. Only for OATs, pockets were directly identified with the FLAPsite tool as implemented in the FLAP software (Baroni et al. 2007). All pockets for the investigated set of proteins are shown in Fig. S1. The OPLS4 force field was employed for receptor grid generation, whilst OPLS3.5 was used for RD and IFD (Jorgensen and Tirado-Rives 1988; Roos et al. 2019). Most settings were kept as standard for all calculations, that is, 20 Å for the cubic box side, amides and ring conformations exploration, energy-based filtering for the resulting poses. Residue flexibility, explored during the Prime step in the IFD, was increased from 5 to 8 Å away from the centre of the pocket to account for longer molecules and their possible interactions with additional residues. An exception was made for L-FABP, a well-documented target, whose single pocket is known to be bound by different PFAS (Woodcroft et al. 2010; Sheng et al. 2016). To properly validate our in silico data with respect to literature information, we applied and evaluated different docking methodologies on L-FABP, one of the most studied and documented FABP. In particular, we performed unconstrained standard docking, H-bond constrained docking to Ser39 and Arg122, positional constrained docking centred on the myristic acid carboxylic carbon (PDB ID 3STK was used as reference in this last case). We gathered available computational and experimental studies reported in literature about L-FABP:PFAS interaction and built a small dataset of affinity data (Zhang et al. 2013a; Sheng et al. 2016; Cheng and Ng 2018; Cheng et al*.* 2021). Dissociation constants (Kd) values were converted to their corresponding free energy (ΔG) values. The estimated binding free energies obtained with the different docking procedures were compared to literature affinity data. The docking method showing the lowest deviation was then used as our standard for the simulations performed on the other FABP members. In particular, positional constrained docking proved to be the most consistent approach. As previously mentioned, we should remind that PFAS chain rigidity is markedly different with respect to the corresponding hydrogenated compounds (fatty acids), and is not correctly accounted for by standard molecular mechanics force fields (Borodin et al. 2002). This might allow some discrepancies between computational simulations and experimental findings.

### Molecular dynamics

Gromacs 4.6.1 (Hess et al. 2008; Abraham et al. 2015) package was used to run plain and unbinding MD simulations. TIP3P was used as the water model. The solvated system was minimised using 5000 steps of steepest descent. The Verlet cutoff scheme, the Bussi − Parrinello thermostat, LINCS for the constraints (all bonds), and the particle mesh Ewald for electrostatics with a short-range cutoff of 11 Å, were applied. Four equilibration steps were performed: from 0 °K to 100 °K, from 100 °K to 200 °K, from 200 °K to 300 °K lasting, respectively, 0.1, 0.1 0.1 ns, lastly a final equilibration at 300 °K lasting 1 ns. The first three equilibrations were performed in NPT ensemble, whilst the last was run in NVT. In the two first equilibration steps, harmonic positional restraints were applied to the backbone of the protein with a spring constant of 1000 kJ/(mol·Å2). The production run was carried out in the NVT ensemble at 300 °K without any restraint for 200 ns, at an integration step of 2 fs. MD production on HSA was run for 500 ns, considering the dimension of the protein and the number of pockets. MD setup and trajectory cleaning were performed using the BiKi LifeScience suite (Decherchi et al. 2018). The ligand was parameterized using the *ab-initio* RESP-DFT-charge-fitting methodology and converted into the GROMACS format using the acpype tool. The analyses of the MD trajectories were performed with the GROMACS package, and Bridge2 (Siemers et al. 2019) software was applied for further H-Bond analysis and visualisation.

In the specific case of HSA, the 7AAI structure was submitted to MD simulations, after removing everything but the four PFOA molecules in the FA4, FA6, FA7 and cleft pockets, and a molecule of myristic acid (MYR) bound in FA3 and known to be important for the stability of FA4-bound ligands. MYR in FA3 was also maintained in all docking analyses. The same HSA structure was used to build an HSA:4cC6O4 complex, upon removal of the PFOA molecules and the docking of four cC6O4 molecules in the same sites. Specifically, only cC6O4 RS isomer has been reported in the simulations, since only slight differences have been observed for the other stereoisomers.

In the case of TTR, MD simulations were run in triplicate for 200 ns on a chimeric protein structure binding the natural ligand T4 in one site (site A), and the PFAS, PFOA or cC6O4, of interest in the other (site B), to take account of the possible negative cooperativity.

Unbinding simulations were carried out on TTR-PFOA and TTR-cC6O4 complexes. The protein − ligand complexes were used as a starting point for MD simulations performed in a GROMACS 4.6.1 version customised to run scaled molecular dynamics implemented by BiKi Life Sciences (Mollica et al. 2015, 2016). To get statistically significant results, 20 *replicas* of 30 ns each have been carried out, using a softened potential by a factor of 0.4.

### Chemicals

cC6O4, as ammonium salt (CAS RN 1190931-27-1, purity: ≥ 97.5%), was provided by Solvay Specialty Polymers Italy S.p.A., perfluorooctanoic acid (PFOA, CAS 335-67-1) was purchased from Merck Life Science S.r.l. Stock solutions of cC6O4 and PFOA were prepared by dissolving the compounds in water at a concentration of 1 mg/ml.

### PFOA/cC6O4 binding to BSA

#### Dialysis assays

For dialysis experiments, Hank’s balanced salt solution (HBSS, pH adjusted to 7.4) was purchased from Sigma-Aldrich (St. Louis, MO, USA), and used as buffer. Bovine serum albumin (BSA) was purchased from Sigma-Aldrich (St. Louis, MO, USA) and used at a concentration of 3 g/l in HBSS. The compounds were studied at a concentration of 1 μg/ml, obtained by diluting the aqueous stock solutions in HBSS, to avoid the oversaturation of protein binding sites. As dialysis system, we used Thermo Scientific™ Slide-A-Lyzer™ MINI Dialysis Devices, with cellulose membrane 3.5 K MWCO. Dialysis equilibrium experiments were performed in parallel on reference and on measurement cells. Reference system consisted of 0.5 ml solution of compound (1 μg/ml in HBSS) added in the MINI Dialysis Devices (donor Reference chamber, dR) and 1 ml of HBSS added in the receiving chamber (rR). Measurement system was 0.5 ml solution of compound (1 μg/ml in HBSS) added in the MINI Dialysis Devices (donor measure chamber, dM) and 1 ml of BSA solution (3 g/l in HBSS) introduced in the receiving chamber (rM). The donor and receiving compartments were assembled and were taken at 37 °C ± 0.5 °C under gentle shaking. After 72 h of incubation, the compounds were quantified in BSA free chambers (dR, rR, dM) by UPLC–MS analysis (see below, compounds quantification). PFAS binding to BSA was then calculated with the following equation (considering that at the equilibrium dM concentration is equal to rM concentration):

$$\% \, bound \, = \, 100 \, - \, \left[ {100{{ \cdot }}conc.dM{{ \cdot }}vol.rM} \right)] \, / \, \left( {conc.dR{{ \cdot }}vol.dR \, + \, conc.rR{{ \cdot }}vol.rR \, - \, conc.dM{{ \cdot }}vol.dM} \right)$$

where *conc.dM, conc.dR, conc.rR* = concentration of compounds (μg/ml) measured by UPLC–MS analysis

vol.rM, vol.rR = 1 ml

vol.dM, vol.dR = 0.5 ml

The recovery of the dialysis process, determined with the help of the reference chamber, was evaluated to discover whether the compound was lost during process, considering PFAS tendency to adsorption.

$$\% {\text{Recovery }} = { 1}00{{ \cdot }}\left( {conc.dR{{ \cdot }}vol.dR + conc.rR{{ \cdot }}vol.rR} \right)/vol.dR$$

### Compound quantification

#### Sample preparation

Samples from dialysis were filtered over 0.45 μm nylon syringe filter, transferred into the suitable vials and analysed by UPLC–MS/MS, without any other pre-treatment. To confirm that nylon filters (0.45 μm) did not retain the per-fluorinated compounds, standard samples dissolved in cell culture medium were analysed before and after filtration. No filter retention was observed.

#### Standard preparation

cC6O4 and PFOA standard solutions were prepared diluting aqueous standards in HBSS (pH = 7.4) to take into account the possible matrix effect. Calibration curve of compounds was obtained over a concentration range of 0.05–2.5 μg/ml (*r*^2^ > 0.99).

#### UPLC–MS/MS analysis

Quantification of analytes was carried out in MRM modality on UPLC-TQD system (Waters Acquity-TQD), using Adamas C18-X-Bond column (2.1 × 50, 1.8 µm) maintained at 40 °C, and following a previously reported method (Bruno et al. 2022). Briefly, elution was carried out in gradient mode: aqueous solution of AcONH_4_ 10 mM (A), acetonitrile (B), at 0.200 ml/min. Gradient profile: (time, B%) 0.0, 5; 6.2, 40; 7.0, 50; 8, 80; 11, 80). cC6O4 was quantified using the transition 338.90- > 179.00 (m/z) with collision energy of 5 eV; the transition 338.90−  > 85.00 (m/z), with collision energy of 22 eV, was used to check the identity of the compound. PFOA was quantified using the transition 313.00−  > 269.00 (m/z) with collision energy of 9 eV; the transition 313.00−  > 119.00 (m/z), collision energy of 21 eV, was used to check the identity of the compound.

## Results and discussion

The possible interaction of the PFAS series with the listed protein targets has been estimated by means of molecular docking simulations performed with Glide (Schrodinger 2022–4). The procedure returns, for each possible interaction, a score measured in kcal/mol, which estimates the binding free energy as a sum of contributes, according to Friesner et al. (2006).

Then for specific cases, MD simulations were run, to verify the binding stability, in particular in the case of highly flexible proteins, whose binding site might exhibit different residue orientation than those obtained through docking simulation, and where ligands might assume unexplored or additional poses. MD simulations were also run to compare the stability of legacy PFAS in the considered targets, with respect to novel PFAS, expected to have lower affinity and lower residence time (Bruno et al. 2022).

### Human serum albumin

HSA is known to be the PFAS primary vector in the human body (Forsthuber et al. 2020; Crisalli et al. 2023), thanks to PFAS structure being like that of fatty acids (Gao et al*.* 2019; Alesio et al. 2022).

Multiple possible pockets, named fatty acid (FA) pockets, have been identified by means of X-ray diffraction and docking simulations, but only few of them are known to bind PFAS with varying affinity (Maso et al. 2021; Perera et al. 2022; Moro et al. 2022). In particular, four pockets have been found occupied by PFAS in PDB ID 7AAI (Maso et al. 2021; Alesio et al. 2022), but further structural analysis and literature search revealed that up to five additional pockets could accommodate molecules as big as myristic acid.

**Fig. 1:**
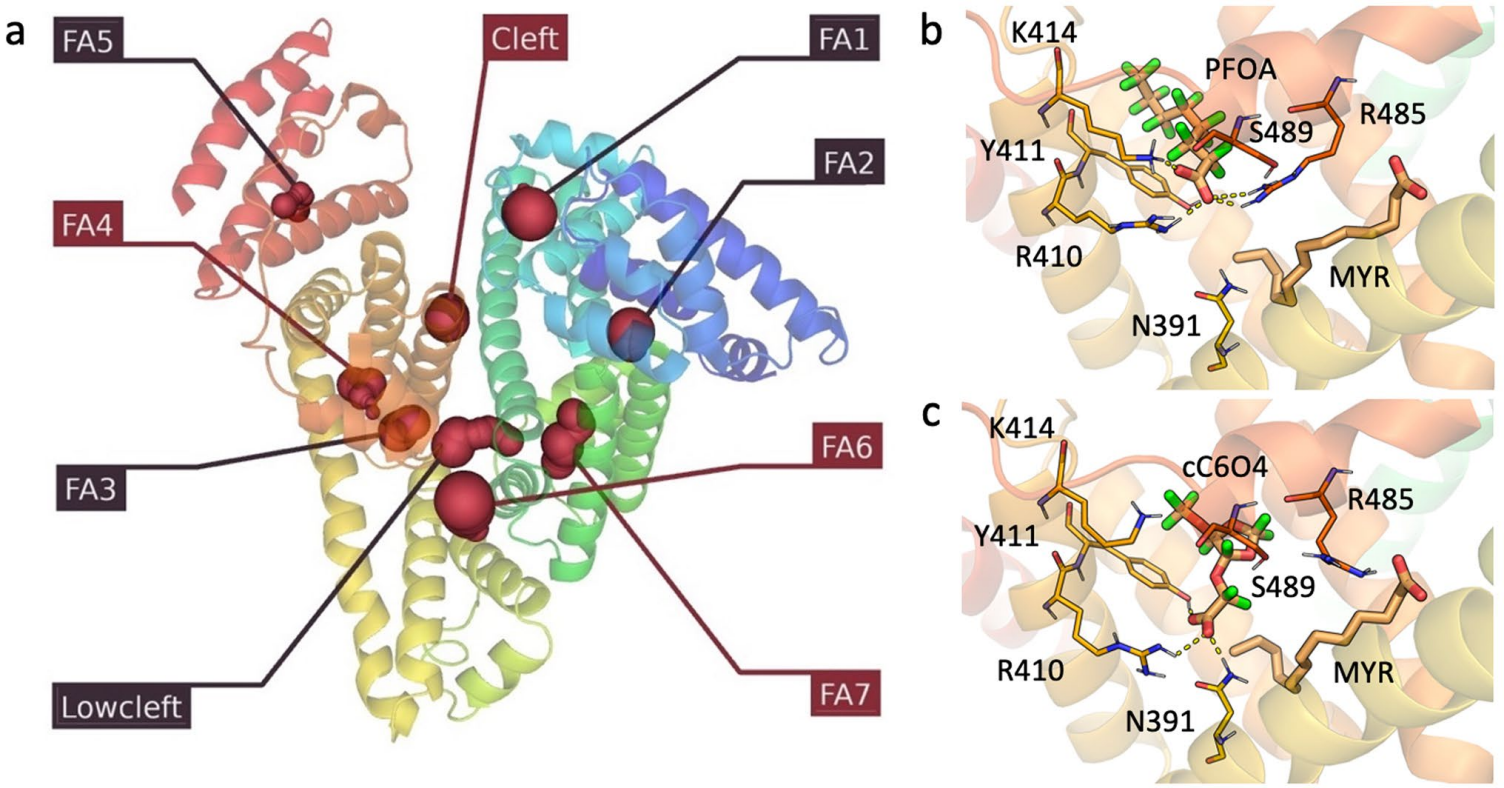
**a** HSA pockets shown as red contours (PDB ID 7AAI). The protein is shown as cartoon. The red labelled pockets are occupied by PFOA. **b** PFOA docked in FA4 pocket. **c** cC6O4 docked in FA4 pocket. Ligands and lining residues are shown as capped sticks, H-bonds as yellow dashed lines

We, thus, decided to expand our docking calculations to all nine pockets (Fig. 1).

As can be observed by the docking scores reported in Fig. S2, narrower pockets FA1, FA2, FA4 and FA5 (Zunszain et al. 2003; Moro et al. 2022) easily accommodate linear PFAS, whilst larger ones also allow bulkier PFAS to bind, even if with overall low affinities. PFOA, known as one of the best binders in FA4, resulted in a minimum of − 13.4 kcal/mol, whilst slightly smaller or larger molecules, like PFHxA and PFNA, have higher scores and therefore lower affinity. A similar trend was observed for sulfonic acids, with a minimum of − 10.8 kcal/mol for PFDS. PFBA constitutes a peculiar case in HSA, and in other targets. Indeed, with respect to longer PFAS likely involved in a higher number of hydrophobic contacts, PFBA showed low scores, that is high affinity, in different pockets, despite the short hydrophobic tail. In general, the highest affinity site for PFOA, i.e. FA4, shows a trend similar to the results reported in literature (Moro et al. 2022; Crisalli et al. 2023): PFOA and PFHxA show the highest affinity, whilst short-chained cC6O4 and GenX present lower affinity than linear, longer PFAS. PFOO, which lacks a functional group, shows affinities of about -7 kcal/mol in different pockets, but very few poses. This is reasonably given to the absence of polar interactions and is indicative of an unfavourable binding.

Overall, the results obtained for the other pockets are in line with those described for FA4, with 8–10 carbon long PFAS showing the most promising interaction, few to no poses for longer molecules, i.e. from 12 to 18 C atoms, and PFBA being present and with good, predicted affinity, despite the absence of experimental indication about a possible interaction with HSA. Interestingly, less hydrophobic, and more branched novel PFAS show, in general, less stable interactions, likely suggesting a lower probability of being bound and transported by HSA. No appreciable differences were observed amongst the analysed PFAS when docked in low affinity sites, as FA6 or cleft.

The X-ray structure of HSA complexed with four PFOA molecules (PDB ID 7AAI; sites FA4, FA6, FA7, cleft) was then submitted to a 500 ns-long plain MD simulation. To compare PFOA binding and stability in HSA to that of the new generation PFAS cC6O4 (Bruno et al. 2022), we built a construct of HSA complexed with four cC6O4 molecules, obtained by IFD, and submitted it to an equal long MD simulation. The RMSD calculation showed the protein remain stable and compact in both simulations (Figure S3). The stability of the different ligand copies in the four pockets is shown in Fig. S4, where the ligand RMSD, calculated by centring the least square fitting on the pocket residues, and the corresponding moving mean are reported, to better show the ligand movement in and out the pockets.

Both PFOA and cC6O4 appear to be quite stable in FA4, as the RMSD profiles do not present significant variations (Figure S4a). This is also confirmed by the H-bond persistency, showing the maintenance of the interaction with positively charged residues for more than 90% of the simulation time, as shown in detail in Fig. S5. A similar trend can be observed for FA7 pocket, even if, on average, cC6O4 presents slightly larger fluctuations, indicative of a less stable interaction (Figure S4c). On the contrary, the ligands show a completely different behaviour in the solvent-exposed and low-affinity pocket FA6, where PFOA does not undergo significant variations, whilst cC6O4 at ~ 200 ns reports a huge RMSD increment (Figure S4b). This change is given to an abrupt loose of contact with Arg207, and to the subsequent movement of the molecule along the protein surface towards Lys349 (Figure S5b), with which it forms a stable H-bond. The case of the cleft pocket is quite peculiar: it is a largely solvent-exposed pocket and there is only one paper reporting about it being occupied by a ligand (Maso et al. 2021). Both PFOA and cC6O4 are quite unstable in the pocket, suggesting this site can be likely occupied only at very high ligand concentration. The number of contacts between the ligands and any atom within 0.6 nm are reported in Fig. S6. Overall, MD simulations seem to suggest PFOA can be transported by HSA with higher probability than cC6O4. This is in particular highlighted by all performed analyses (Figs. S4–S6), showing that, in all pockets except the cleft, PFOA is more stable than cC6O4. As described in literature (Crisalli et al. 2023), this difference can be mainly attributed to entropic effects arising from different tail length, whilst enthalpic ones, like H-bonds, remain reasonably constant.

These observations have been experimentally confirmed by measuring the percentage of PFOA and of cC6O4 binding to bovine serum albumin (BSA) through dialysis equilibrium experiments. The percentage of PFOA and cC6O4 bound to BSA corresponds to 82% and 62%, respectively (see Table S2). These experiments also highlighted a degree of absorption of compound PFOA (recovery 85%) on the plastic material of dialysis devices, phenomenon not observed in the case of cC6O4 (recovery 99%). These results suggest that novel PFAS should have a lower probability of binding albumin with respect to legacy PFAS.

### Transthyretin

TTR presents two identical and symmetrical binding sites (AC and BD), in which the natural substrate thyroxine (T4) is stabilised by hydrophobic contacts with most of the residues lining the pockets, and by electrostatic interaction with Lys15 and Lys15’ (PDB ID 2ROX, (Wojtczak et al. 1996). Similar contacts are made by PFOA (PDB ID 5JID (Zhang et al. 2016)). According to Purkey et al. 2001, and Tomar et al. 2012, TTR binds thyroxine T4 and other ligands with a stoichiometry lower than 2 in physiological conditions. Considering this indication, the negative cooperativity suggested by Neumann et al. (Neumann et al. 2001), and the fact that TTR is present in the serum at a concentration much higher than PFAS (Ingenbleek and Bernstein 2015; Göckener et al. 2020), we hypothesised that in standard conditions, only one PFAS molecule at a time would be able to bound TTR. We, thus, docked the PFAS series in only one of the two pockets (Figure S1b), assuming the same results could be expected for the second site, considering the two are basically identical (Dharpure et al. 2023).

When re-docked in TTR, PFOA superimposed very well to the co-crystallised ligand (Figure S7), thus further validating the docking procedure, and showed a score about -7 kcal/mol. The latter corresponds to one of the lowest (Figure S8), even if there are not extremely significant differences amongst the considered PFAS. In general, the profound hydrophobic channel of TTR easily accommodates PFAS carbon chain, whilst the two gating lysines, Lys15 and Lys15’, stabilise the negatively charged moiety by means of salt bridges. Specifically, medium-length PFAS, (6–10 C atoms) show a mediocre affinity for TTR, with PFDA resulting in a minimum of -7.3 kcal/mol, whilst PFNA and PFOA differ by less than 0.5 kcal/mol (− 7.1 and − 7.0 kcal/mol, respectively). Smaller or bigger molecules have slightly higher scores (e.g. − 5.7 kcal/mol for PFBA), and therefore slightly minor affinities for this target. cC6O4 returned a score of − 5.6 kcal/mol, more similar to low affinity molecules than medium ones. These results agree with those of (Ren et al. 2016), who reported higher affinity for PFOA (IC_50_ equal to 378 nM) and lower for PFDA and PFNA (1623 and 1977 nM, respectively).

MD simulations were carried out with the intent of investigating legacy and novel PFAS stability in TTR, again taking as reference PFOA and cC6O4. Specifically, 200 ns-long MD was performed in triplicate, for a total of 600 ns. As shown by RMSD calculation (Figure S9), the protein is stable throughout the whole dynamics, only showing higher flexibility in loops and terminal regions for each monomer. Interestingly, whilst PFOA maintains its pose favourably interacting with the hydrophobic residues lining the pocket and H-bonding the two gating Lys15 and Lys15’, cC6O4 shows higher fluctuations, meaning a reduced stability in the pocket. Moreover, one in three *replicas* shows displacement of cC6O4 exiting the funnel-shaped binding pocket (Figure S10). The lower number of contacts that cC6O4 forms with apolar residues, compared to PFOA, is mainly due to its shorter and cyclic chain. In addition, the stronger interactions between the ligand carboxylate and the positively charged amino groups of Lys15 and Lys15’ may help to explain the ligand movement towards the exterior of the pocket. T4, which is in pocket A (Figure S1b), shows different stability depending on whether the protein is complexed with cC6O4 or PFOA. In the former case, T4 RMSD values are low (less than 0.1 A) and conserved in the three replicates. In addition, T4 favourably H-bonds to Thr119, a residue located in the innermost portion of the pocket, thus explaining its higher stability in the binding site. On the other hand, T4 in the TTR:PFOA/T4 complex shows higher fluctuations and a reduced H-bond occupancy with Thr119, due to the increased distance between the ligand and the residue (Figure S11), meaning a lower stability in the pocket. The observation of imbalance between the higher affinity of PFOA for site B and the lower stability of T4 in pocket A, as well as the worse affinity of cC6O4 and the better stability of T4, could be explained by negative cooperativity, (Ferguson et al. 1975; Neumann et al. 2001; Tomar et al. 2012; Haupt et al. 2014).

Finally, additional unbinding simulations were carried out on both complexes to rank and classify the ligands according to their residence time in the binding site. Simulations resulted in an unbinding time of 10.8 (± 1.82) ns for PFOA, and 8.5 (± 1.69) ns for cC6O4. Due to the proportional correlation between the residence time and the binding affinity, the lower values for cC6O4 may assess a lower affinity for TTR compared to PFOA.

### Thyroxine binding protein

TBG has an even lower serum concentration than TTR (1.1–2.1 mg/dL), but a much higher affinity for T4 and T3 (K_a_ equal to 10^10^ and 2·10^8^ (M^−1^), respectively (Refetoff 2000)). It presents a single and quite large binding site, characterised by the presence of two arginines. Arg378 forms a salt bridge with the substrate carboxylic moiety, whilst Arg381 forms a π–cation interaction with the substrate aromatic ring (Ren et al. 2016; Figure S12). Even if no crystallographic information is available about PFAS interacting with TBG, binding experiments showed a distinct drop in affinity after performing site mutagenesis on Arg378 or Arg381. We, thus, guided the docking pose selection according to the capability of PFAS carboxylic, sulfonic or hydroxyl group to H-bond to one of the arginine residues.

Molecular docking returned results quite similar to those reported for TTR, with a general poor affinity and no significant variations, apart for the longer molecules, which did not retrieve any pose (Figure S13). In particular, the lowest values were obtained for the R stereoisomer of ADONA and PFUnA (− 6.7 kcal/mol and − 6.6 kcal/mol, respectively). Medium-length molecules gave slightly lower affinities, with PFOS giving the minimum for the sulfonic acid category (− 6.1 kcal/mol). Less favourable values were obtained also for GenX (-5.0 kcal/mol) and for cC6O4, giving a minimum of − 5.7 kcal/mol, but with only a handful of poses. The docking pose of PFOA and cC6O4 in TBG is reported in Fig. S14.

However, the few available experiments showed a different trend, with the longest molecules, i.e. PFODA, telomers, being the only with a significant affinity towards TBG (Ren et al. 2016).

MD simulations of the TBG:PFOA complex showed conserved interactions between the carboxyl group and only one of the two arginines of interest, Arg381, establishing additional H-bonds with Ser23, Ser24 and Lys270, whilst the hydrophobic end of PFOA faced towards Arg378. The docking pose appeared to be conserved over the three dynamics, with main fluctuations related to the fluorinated carbon chain probably due to its reduced size and, thus, inadequate filling of T4 pocket. In the case of the TBG:cC6O4 complex, two out of three *replicas* reported a similar H-bond pattern to that shown for PFOA. However, the cC6O4 pose appeared to be less conserved since the molecule adopted a different orientation in the third *replica*, with the carboxylic group exposed towards both Arg378 and Arg381 and the terminal per-fluorinated carbon directed on the other side of the binding site. Analogously to PFOA, cC6O4 showed a discrete degree of movement due to its small and compact size not completely filling the binding pocket (Figures S15, S16).

### Fatty acid binding proteins

PFAS are overall good ligands for FABPs (Cheng et al. 2021; Fenton et al. 2021; Crisalli et al. 2023). Given their similarity to fatty acids, they assume a similar binding mode in FABP binding sites, forming hydrophobic interaction with the many lipophilic residues lining the pocket and electrostatic contacts with conserved arginine in all isoforms. However, the different pocket size in FABPs plays a role in docking results. As a reference, we considered and reported in Fig. S17 the crystallographic pose of palmitic acid in L-FABP (PDB ID 3STK). The different FABP pocket size is shown in Fig. S1d-h. In particular, amongst the FABPs included in the present study (L-FABP, I-FABP, H-FABP, A-FABP, PmP2), L-FABP has the biggest pocket (Smathers and Petersen 2011) and is able to accommodate PFAS with up to 12 carbon atoms (Zhang et al. 2013a). This observation has been confirmed by our simulations, showing the lowest value (− 13.0 kcal/mol), and the highest affinity, for PFDoDA in L-FABP (Figure S18a), and higher values for shorter compounds. PFPeA and PFBS showed the highest score values for carboxylic and sulfonic acids, respectively.

A similar trend can be observed for I-FABP, for which PFDoDA gave, again, the lowest score of − 13.6 kcal/mol (Figure S18b). Very good scores, in this case, were obtained also for PFNA and, in general, for shorter molecules as PFOA, in agreement with the reduced dimension of I-FABP binding site, likely able to properly host even smaller compounds. PFBA and PFHpS showed the highest scores for carboxylic and sulfonic acids, respectively. For both L-FABP and I-FABP, ADONA and GenX presented similar score values, with the minimum ranging in the − 12/− 10 kcal/mol range, and numerous poses, indicative of a favourable interaction. cC6O4, on the other hand, reported higher scores: − 9.4 kcal/mol in L-FABP and − 8.1 kcal/mol in I-FABP, with fewer poses when compared to the other two perfluoroethers. Similar trends have also been also reported by (Cheng et al. 2021).

Even if with higher values, also H-FABP seem to properly host linear carboxylic and sulfonic acids (Figure S18c). PFDoDA presented a minimum score of − 11.8 kcal/mol and was the biggest molecule able to dock inside this pocket, Smaller molecules showed increasingly higher scores, that is, lower affinities. Sulfonic acids demonstrated a downward trend with PFOS reaching − 8.0 kcal/mol docking score, whilst fluorotelomers gave comparable results. Amongst novel PFAS, ADONA reached the best affinity of − 10.2 kcal/mol, GenX around − 8.7 kcal/mol and cC6O4 of − 9.2 kcal/mol. It is also important to note that both isoforms SS and RR of cC6O4 gave a single docking pose, whilst other novel PFAS returned multiple poses.

A distinguishing case in our study is represented by A-FABP (Figure S18d). Being the protein pocket smaller with respect to other isoforms, many carboxylic acids were not processed by the docking algorithm, or returned a very few poses, as previously mentioned, indicative of unfavourable binding (Smathers and Petersen 2011). A downward trend is clearly observable for sulfonic acids and hydroxyl compounds, whilst novel PFAS gave good affinities, with cC6O4 showing the lowest minimum (− 9.4 kcal/mol) and numerous docking poses. Indeed, A-FABP is the only target in our study to show a higher affinity for cC6O4 than for other PFAS.

On the other hand, PmP2 is much more in line with L-FABP, I-FABP and H-FABP (Figure S18e). Linear PFAS bind with ease in these pockets and have a docking pose similar to fatty acids, with a minimum energy found for PFNA equal to − 11.4 kcal/mol. For GenX, no docking pose has been identified for this target, whilst ADONA and cC6O4 showed minimum values about − 9 kcal/mol (− 9.4 and − 9.2 kcal/mol, respectively).

No poses were found for PFOO and PFTeCO in any of the analysed FABPs. Again, this can be reasonably attributed to the impossibility of forming effective interactions with the positively charged arginines lining the binding site.

MD simulations were run for PFOA and cC6O4 complexed to L-FABP, being, by far, the most studied FABP, at least concerning PFAS binding (Zhang et al. 2013a; Sheng et al. 2016, 2018; Cheng and Ng 2018; Gao et al. 2019). In particular, similarly to TTR, we run two 200 ns-long* replicas* for both the L-FABP:PFOA and L-FABP:cC6O4 complexes. The RMSD calculated for the protein shows the conformation is stable in both cases (Figure S19). Differently, whilst the RMSD for PFOA, with fitting on the pocket residues, shows a stable behaviour after a conformational change at 70 ns in both *replicas*, the RMSD calculated for cC6O4 shows larger variability, suggesting a less stable binding (Figure S20).

### Organic anion transporters

**Fig. 2:**
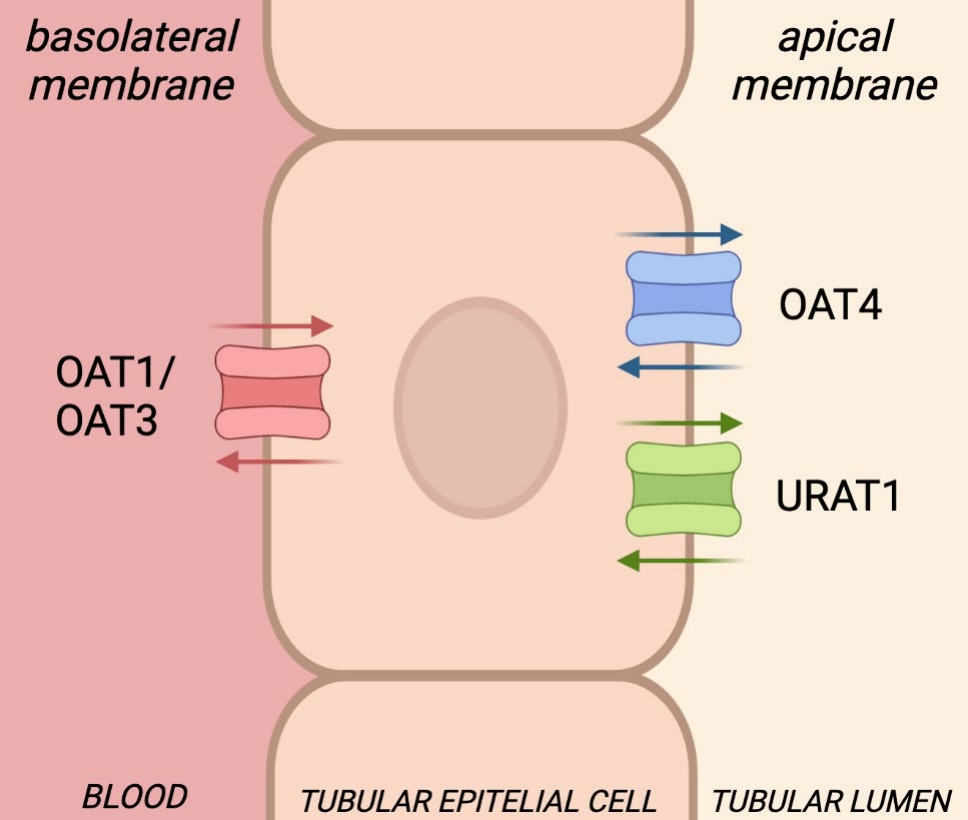
Cellular localisation of uric acid transporters

As the major excretion pathway of PFAS is through urine (Ng and Hungerbuehler, 2015; Fu et al. 2016; Göckener et al. 2020; Fenton et al. 2021; He et al. 2023), whilst secondary pathways are more gender specific (Zhang et al. 2013b), we focused on the possible interaction of PFAS with kidney transporters, i.e. OATs, having uric acid as main physiological substrate. OATs are expressed on both sides of epithelial kidney cells, with similar targets but opposite function: OAT1 and OAT3 (amongst others) mediate chemical excretion from the blood into the urine, whilst OAT4 and URAT1 reuptake the same ligands, from the urine back into the bloodstream (Fig. 2).

These antiporters display multiple conformations, each having a specific role in cell ligand homeostasis. In particular, the *outward* form corresponds to the transporter (on either cell membrane, that is, apical or basolateral) open towards the extracellular side, whilst the *inward* state presents the transporter open towards the cytosol. According to the specific transporter function and localization, both *outward* and *inward* conformations can indicate either PFAS excretion or reabsorption. We, thus, considered both states in our simulations. Whilst an *occluded* conformation also exists, we did not include it in this paper.

OAT1 show a marked difference between *outward* and *inward* states. In the *outward* one, a minimum of − 13.2 kcal/mol is found for PFDA, and less favourable interactions for compound belonging to other classes than carboxylic acids (Figure S21a1,a2). Novel PFAS show affinities comparable to that of sulfonic acids (minimum of − 10.3 kcal/mol for ADONA). cC6O4 offers a few poses, but still with good affinity. In the *inward* conformation, instead, the score distribution is much flatter, with a downward trend centred on PFHxA, and novel PFAS showing lower affinities up to − 4.2 kcal/mol for cC6O4 RS.

OAT3 (Truong et al. 2008; Li et al. 2019; Janaszkiewicz et al. 2023) struggles to accept molecules bigger than PFNA in the *outward* conformation, but still shows good affinities (maximum affinity of − 10.2 kcal/mol for PFPeA; Figure S21b1,b2). Perfluoroethers show a similar trend as for OAT1, but with slightly less affinity for this target, particularly cC6O4, which shows a minimum of − 7.0 kcal/mol for the SS stereoisomer. The *inward* conformation, on the other hand, shows higher affinities across the entire PFAS series, in particular for carboxylic ethers. Whilst ADONA R shows a minimum for the corresponding group (− 10.7 kcal/mol), GenX R and cC6O4 SS have lower but still comparable affinity (− 9.6 and − 9.4 kcal/mol, respectively). This leads to the hypothesis that whilst both OAT1 and OAT3 excrete PFAS with similar lengths and functional groups, OAT1 releases them in the cytosol with much more facility.

The first reuptake transporter we analysed is OAT4. In the *outward* conformation, a trend similar to that of OAT1 and OAT3 can be observed for all PFAS classes, even with a downward trend for both carboxylic and sulfonic acids (PFOA shows a minimum of − 11.3 kcal/mol). Amongst novel PFAS, as usual, cC6O4 shows less affinity than the others and than legacy PFAS (RS, − 7.6 kcal/mol). This point is made more remarkable by the overall low scores of the other stereoisomers (Figure S21 c1, c2). In the *inward* state of OAT4, instead, more poses are found for all classes of PFAS, and the results of PFOA (− 12.0 kcal/mol) are comparable to those of novel PFAS as, specifically, GenX (− 10.2 kcal/mol for S stereoisomer).

In the *outward* conformation, URAT1 has much more sparse results, with a flatter downward trend for carboxylic acids. Carboxylic molecules having more than six carbons display only a couple of poses (minimum of − 10.9 kcal/mol for PFDA), whilst sulfonic ones of similar length are much more numerous (Figure S21d1,d2). GenX and cC6O4 display significantly lower affinity for this conformation when compared to all other PFAS. Lastly, URAT1 showed a U-shaped trend in the *inward* facing conformation, centred on PFHpA (− 11.5 kcal/mol; Figure S21d2). Novel PFAS showed again lower affinities, ranging from − 8.8 to − 3.0 kcal/mol (GenX S and cC6O4 SR, respectively).

Our results are in line with those reported by (Bruno et al. 2022), who found a remarkably lack of interaction for cC6O4 with OATs, which translates to easier excretion through urine for this PFAS, compared to known ones like PFOA and PFOS, which also gave some of the highest affinities for these targets. Indeed, PFOA and PFOS, known to last for years in the human body (Olsen et al. 2007; Zhang et al. 2013b; Fu et al. 2016; Xu et al. 2020; Fenton et al. 2021; Fustinoni et al. 2023), give lower docking scores, and therefore better interactions with these targets. On the contrary, smaller, bulkier molecules like cC6O4 are in striking contrast and returned less favourable interactions with OAT1 and OAT3. In Figures S22 and S23, we report some docking poses for PFOA and cC6O4 in OAT1 and URAT1, respectively.

## Conclusions

In this work, we reported an in silico approach to predict the binding of a series of PFAS to human proteins involved in chemical binding and transportations. Our simulations showed that novel PFAS have a lower affinity for fatty acids transporters like HSA and FABPs, and also for thyroid-hormone transporters TTR and TBG. This might be associated to an overall lower bioaccumulation when compared to legacy, linear, compounds like PFOA and PFOS. Experiments performed on BSA, taking as reference PFOA and cC6O4, confirmed our in silico simulations.

Excretion and reuptake pathways were also explored, with a particular spotlight on OAT4 and URAT1, which showed good affinity for shorter and bulkier molecules. Given their role as reuptake transporters and the small docking scores shown in our data, we can hypothesise a significantly shorter half-life of bulky fluorocarbon compounds in living tissue with respect to longer and linear compounds. Our results suggest for a possible way PFAS might have to last so long in living tissues, despite their hydrophobicity and lipophobicity. Reuptake proteins mistakenly recognise these molecules as good ligands and re-absorb them again back in the blood stream, where they can circulate bound to serum transporters (He et al. 2023). PFBA, for example, the smallest molecule analysed in this work, is known to last a few days in the serum, with a half-life of 3 days, whilst PFOA and PFOS have a half-life of a few to many years (Olsen et al. 2007; Zhang et al. 2013b; Fu et al. 2016; Xu et al. 2020; Fenton et al. 2021; Fustinoni et al. 2023).

The information obtained with multiple simulation techniques allowed us to suggest that linear PFAS should have a more stable binding to the investigated human proteins, when compared to bulkier and especially shorter PFAS. Therefore, additional computational and experimental studies are needed to better differentiate legacy and novel PFAS behaviour and toxicity in the human body.

### Supplementary Information

Below is the link to the electronic supplementary material.

Supplementary file1 (PDF 18550 KB)

## Data Availability

Any data that are not included in the Supplementary Information will be available on request.
