# Supplementary material for: In silico prediction of the interaction of legacy and novel per- and poly-fluoroalkyl substances (PFAS) with selected human transporters and of their possible accumulation in the human body
Source: Arch Toxicol. 2024 Jun 17;98(9):3035–47. doi: 10.1007/s00204-024-03797-0 (PMC11324760; doi:10.1007/s00204-024-03797-0)
Supplement: Supplementary file 1 — Supplementary file1 (PDF 18550 KB) [file 204_2024_3797_MOESM1_ESM.pdf]

Supplementary Information

In silico prediction of the interaction of legacy and novel per- and poly-fluoroalkyl substances (PFAS) with selected human transporters and of their possible accumulation in the human body.

G. A. Tiburtini[1#], L. Bertarini[1,2#,$], M. Bersani[1], T.A. Dragani[3], B. Rolando[1], A. Binello[1], A. Barge[1], F. Spyrakis[1*]

[1] Dept. of Drug Science and Technology, University of Turin, Turin, Italy
[2] Dept. of Life Sciences, University of Modena and Reggio Emilia, Modena, Italy
[3] Aspidia srl, 20100 Milan, Italy
# These authors contributed equally to the work
$ current address: Dept. of Life Science, University of Modena and Reggio Emilia, Modena, Italy

* Francesca Spyrakis
francesca.spyrakis@unito.it

Table of Content *page*

- **Figures and Table List**

  - **Table S1** Analysed perfluorinated compounds 3
  - **Figure S1 (a-k**) Visual representation of the proteins and related pockets.……... 4
  - **Figure S2 (a-i)** Docking scores for HSA………………………………..……….. 11
  - **Figure S3** Backbone RMSD for HSA:PFOA/cC6O4…..……………………..…… 14
  - **Figure S4 (a-d)** RMSD for HSA:PFOA/cC6O4, fitting on the pockets…......…... 15
  - **Figure S5 (a-b)** Hbond persistency, MD of HSA:PFOA/cC6O4………………… 17
  - **Figure S6 (a-d)** Number of contacts, MD of HSA:PFOA/cC6O4…………..…… 18
  - **Table S2** Binding to BSA, Dialysis Equilibrium experiment……………………. 20
  - **Figure S7** Superposition of X-ray and docked pose for PFOA in TTR…….….…. 21
  - **Figure S8** Docking scores for TTR…………………………………..……….…. 22
  - **Figure S9 (a-b)** Backbone RMSD for TTR:PFOA/cC6O4……….…………….. 23
  - **Figure S10 (a-b)** RMSD for TTR:PFOA/cC6O4, fitting on the pocket…..……... 24
  - **Figure S11 (a-b)** Distance for TTR:PFOA/cC6O4, T4 to Thr119...……………… 25
  - **Figure S12** X-ray pose of T4 in TBG……………………..…………………... 26
  - **Figure S13** Docking scores for TBG………………...……………………... 27
  - **Figure S14 (a-b)** Docked poses of PFOA and cC6O4 in TBG……..…………… 28
  - **Figure S15 (a-b)** Backbone RMSD, MD of TBG:PFOA/cC6O4…...…...……… 29
  - **Figure S16 (a-b)** RMSD of TBG:PFOA/cC6O4, fitting on the pocket.....……… 30
  - **Figure S17** X-ray pose of palmitic acid in L-FABP………………………….. 31
  - **Figure S18 (a-i)** Docking scores for FABPs…………………...……………... 32
  - **Figure S19 (a-b)** Backbone RMSD, MD of L-FABP:PFOA/cC6O4……………... 34
  - **Figure S20 (a-b)** RMSD of L-FABP:PFOA/cC6O4, fitting on the pocket…..…. 35
  - **Figure S21 (a-d)** Docking scores for OATs…………………………………... 36
  - **Figure S22 (a-b)** Docking poses of PFOA and cC6O4 in OAT1 outward...…….. 38
  - **Figure S23 (a-b)** Docked poses of PFOA and cC6O4 in URAT1 outward.....….. 39

Table S1. Analysed perfluorinated compounds database. Each acronym is derived from its common name except for the novel substances, which are usually called by their commercial names. IUPAC names and base structures are provided.

Acronym	Common name	IUPAC name	Structure	*n*=	Acronym	Common name	IUPAC name	Structure	*n*=
PFBA	Perfluoro butyric acid	2,2,3,3,4,4,4-heptafluorobutanoic acid	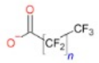	2	PFBS	Perfluoro butanesulfonic acid	1,1,2,2,3,3,4,4,4-nonafluorobutane-1-sulfonic acid	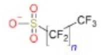	3
PFPeA	Perfluoro pentanoic acid	2,2,3,3,4,4,5,5,5-nonafluoropentanoic acid	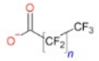	3	PFPeS	Perfluoro pentanesulfonic acid	1,1,2,2,3,3,4,4,5,5,5-undecafluoropentane-1-sulfonic acid	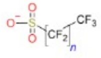	4
PFHxA	Perfluoro hexanoic acid	2,2,3,3,4,4,5,5,6,6,6-undecafluorohexanoic acid	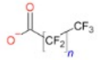	4	PFHxS	Perfluoro hexanesulfonic acid	1,1,2,2,3,3,4,4,5,5,6,6,6-tridecafluorohexane-1-sulfonic acid	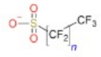	5
PFHpA	Perfluoro heptanoic acid	2,2,3,3,4,4,5,5,6,6,7,7,7-tridecafluoroheptanoic acid	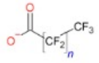	5	PFHpS	Perfluoro heptanesulfonic acid	1,1,2,2,3,3,4,4,5,5,6,6,7,7,7-pentadecafluoroheptane-1-sulfonic acid	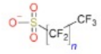	6
PFOA	Perfluoro octanoic acid	2,2,3,3,4,4,5,5,6,6,7,7,8,8,8-pentadecafluorooctanoic acid	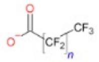	6	PFOS	Perfluoro octanesulfonic acid	1,1,2,2,3,3,4,4,5,5,6,6,7,7,8,8,8-heptadecafluorooctane-1-sulfonic acid	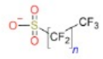	7
PFNA	Perfluoro nonanoic acid	2,2,3,3,4,4,5,5,6,6,7,7,8,8,9,9,9-heptadecafluorononanoic acid	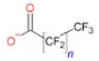	7	PFDS	Perfluoro decanesulfonic acid	1,1,2,2,3,3,4,4,5,5,6,6,7,7,8,8,9,9,10,10,10-henicosafluorodecane-1-sulfonic acid	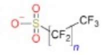	9
PFDA	Perfluoro decanoic acid	2,2,3,3,4,4,5,5,6,6,7,7,8,8,9,9,10,10,10-nonadecafluorodecanoic acid	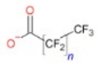	8	6:2FTOH	1,1,2,2-Tetrahydroperfluoro-1-octanol	3,3,4,4,5,5,6,6,7,7,8,8,8-tridecafluorooctan-1-ol	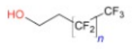	5
PFUnA	Perfluoro undecanoic acid	2,2,3,3,4,4,5,5,6,6,7,7,8,8,9,9,10,10,11,11,11-henicosafluoroundecanoic acid	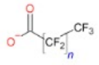	9	8:2FTOH	1,1,2,2-Tetrahydroperfluoro-1-decanol	3,3,4,4,5,5,6,6,7,7,8,8,9,9,10,10,10-heptadecafluorodecan-1-ol	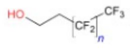	7
PFDoDA	Perfluoro dodecanoic acid	2,2,3,3,4,4,5,5,6,6,7,7,8,8,9,9,10,10,11,11,12,12,12-tricosafluorododecanoic acid	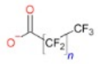	10	PFOO	Perfluoro octane	1,1,1,2,2,3,3,4,4,5,5,6,6,7,7,8,8,8-octadecafluorooctane	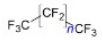	6
PFTrDA	Perfluoro tridecanoic acid	2,2,3,3,4,4,5,5,6,6,7,7,8,8,9,9,10,10,11,11,12,12,13,13,13-pentacosafluorotridecanoic acid	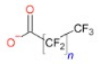	11	PFTeCO	Perfluoro tetracosane	1,1,1,2,2,3,3,4,4,5,5,6,6,7,7,8,8,9,9,10,10,11,11,12,12,13,13,14,14,15,15,16,16,17,17,18,18,19,19,20,20,21,21,22,22,23,23,24,24,24-pentacontafluorotetracosane	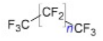	22
PFTeDA	Perfluoro tetradecanoic acid	2,2,3,3,4,4,5,5,6,6,7,7,8,8,9,9,10,10,11,11,12,12,13,13,14,14,14-heptacosafluorotetradecanoic acid	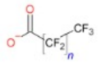	12	ADONA	4,8-Dioxa-3H-perfluoro nonanoic acid	2,2,3-trifluoro-3-[1,1,2,2,3,3-hexafluoro-3-(trifluoromethoxy)propoxy]propanoic acid	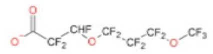	
PFHxDA	Perfluoro hexadecanoic acid	2,2,3,3,4,4,5,5,6,6,7,7,8,8,9,9,10,10,11,11,12,12,13,13,14,14,15,15,16,16,16-hentriacontafluorohexadecanoic acid	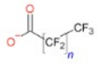	14	GenX	Hexafluoropropylene oxide dimer acid	2,3,3,3-tetrafluoro-2-(1,1,2,2,3,3,3-heptafluoropropoxy)propanoic acid	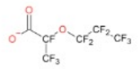	
PFODA	Perfluoro octadecanoic acid	2,2,3,3,4,4,5,5,6,6,7,7,8,8,9,9,10,10,11,11,12,12,13,13,14,14,15,15,16,16,17,17,18,18,18-pentatriacontafluorooctadecanoic acid	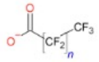	16	cC6O4	/	2,2-difluoro-2-[[2,2,4,5-tetrafluoro-5-(trifluoromethoxy)-1,3-dioxolan-4-yl]oxy]acetic acid	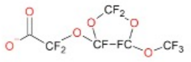	

a

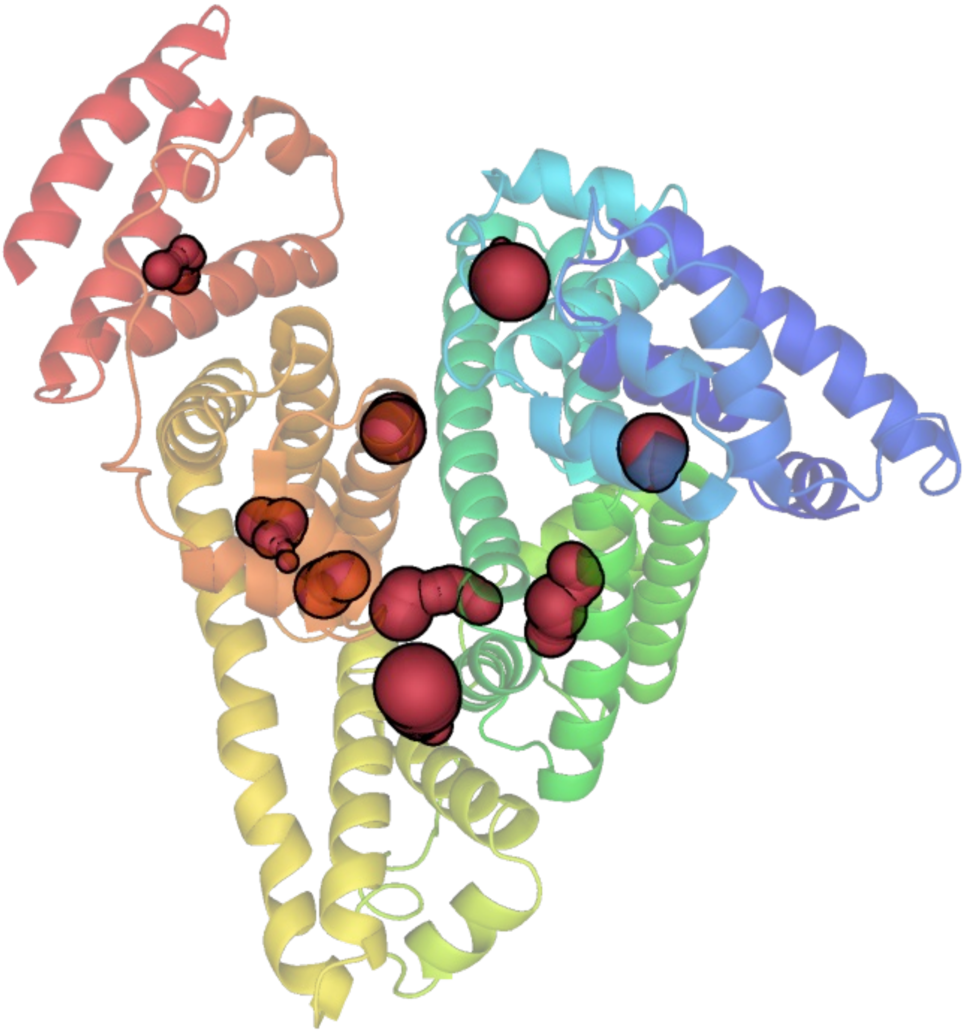

b

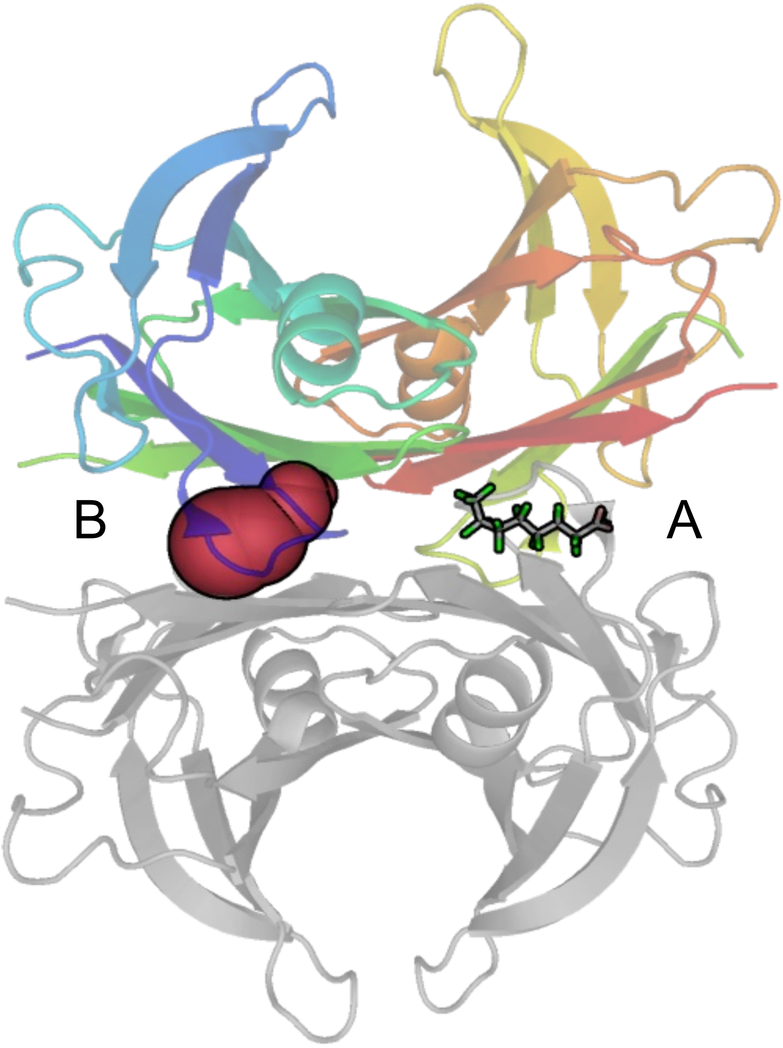

c

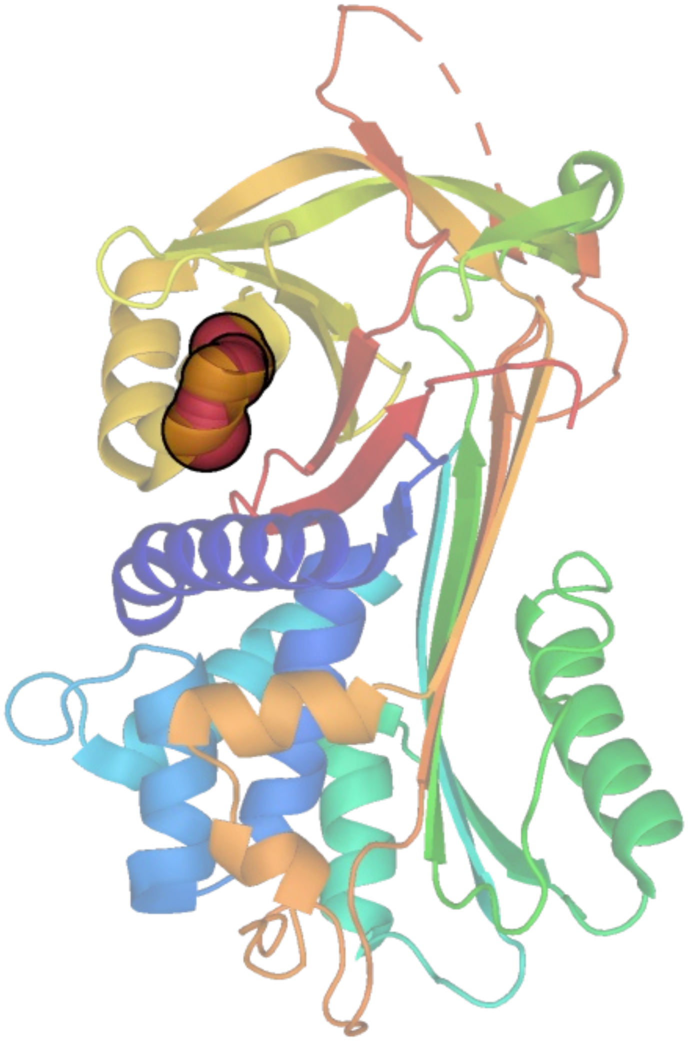

d

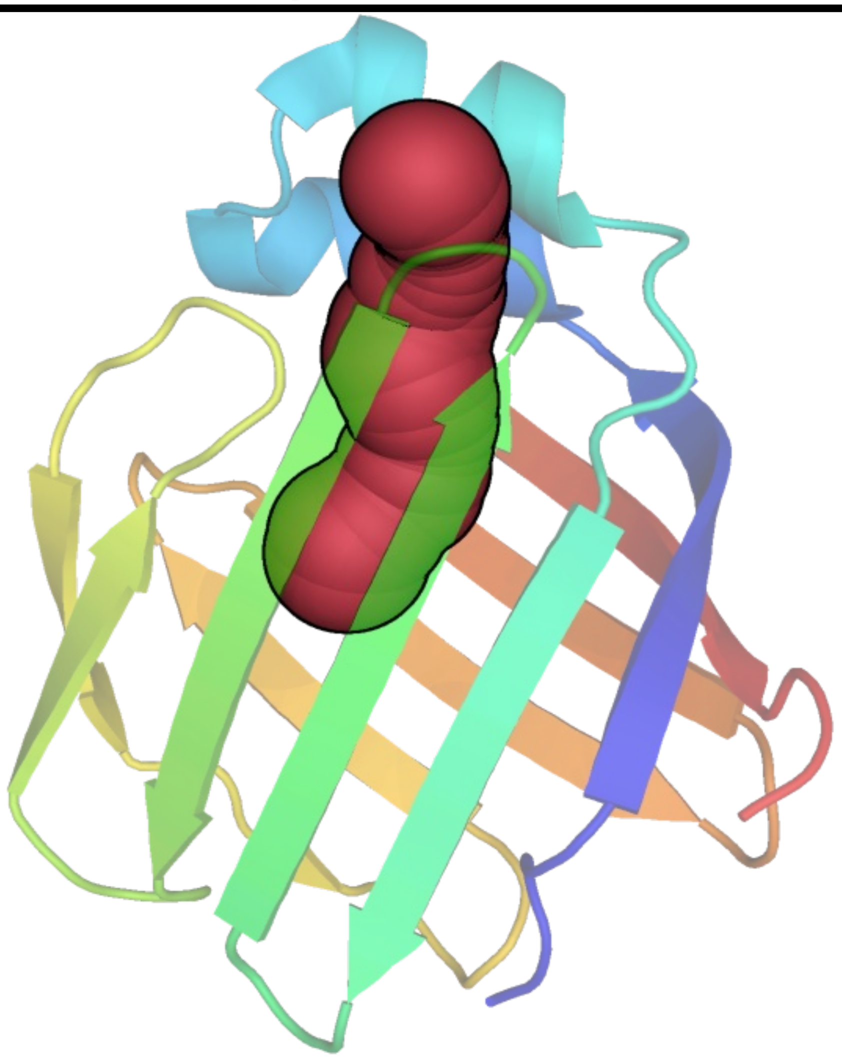

e

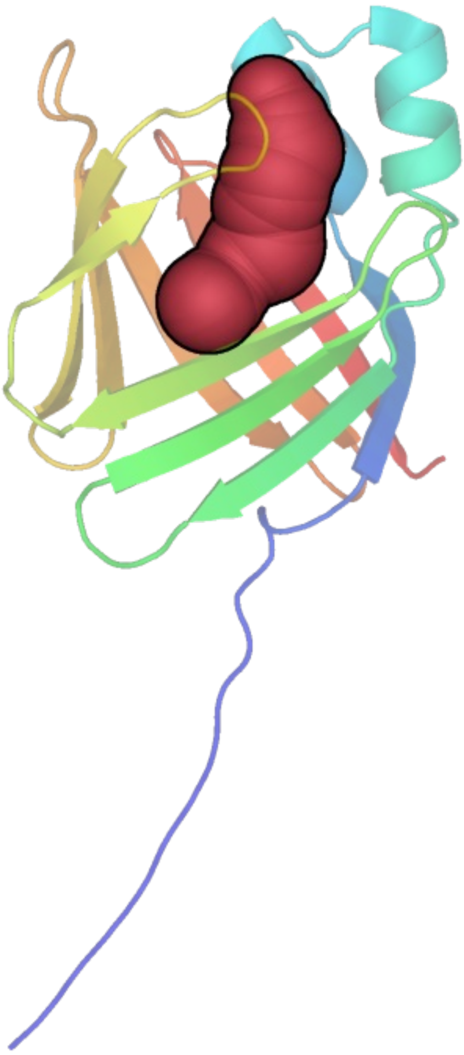

f

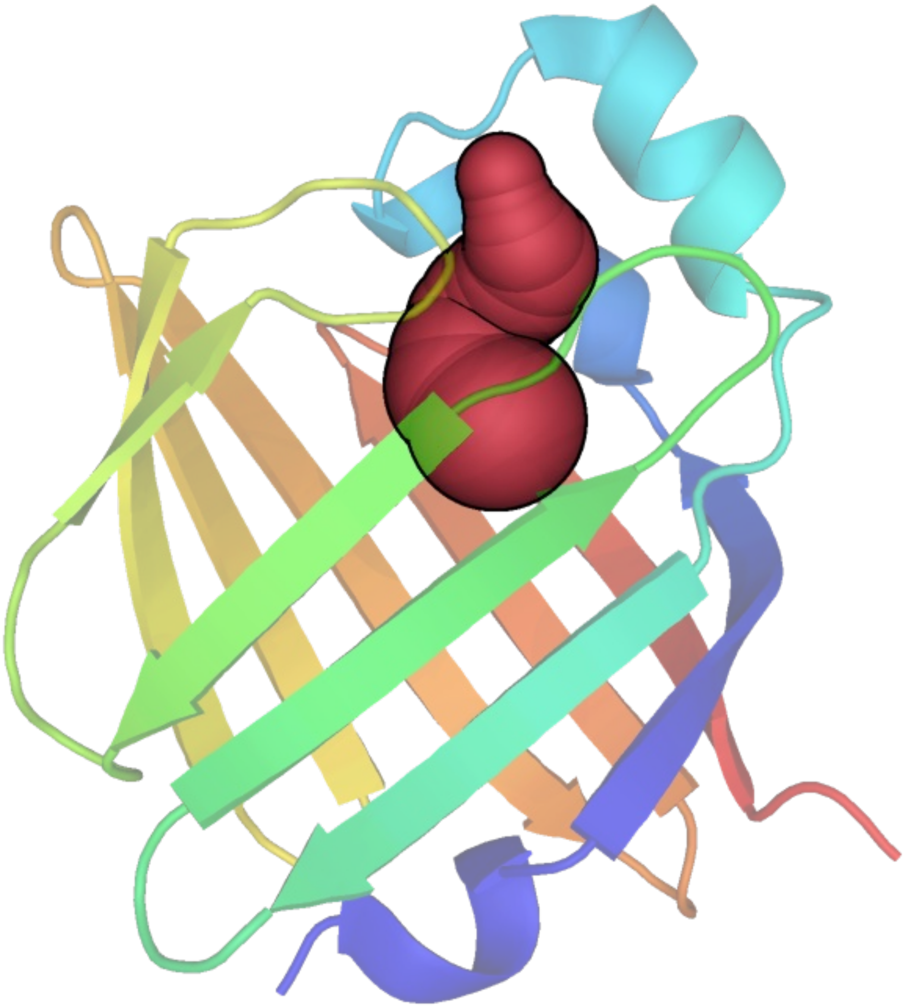

g

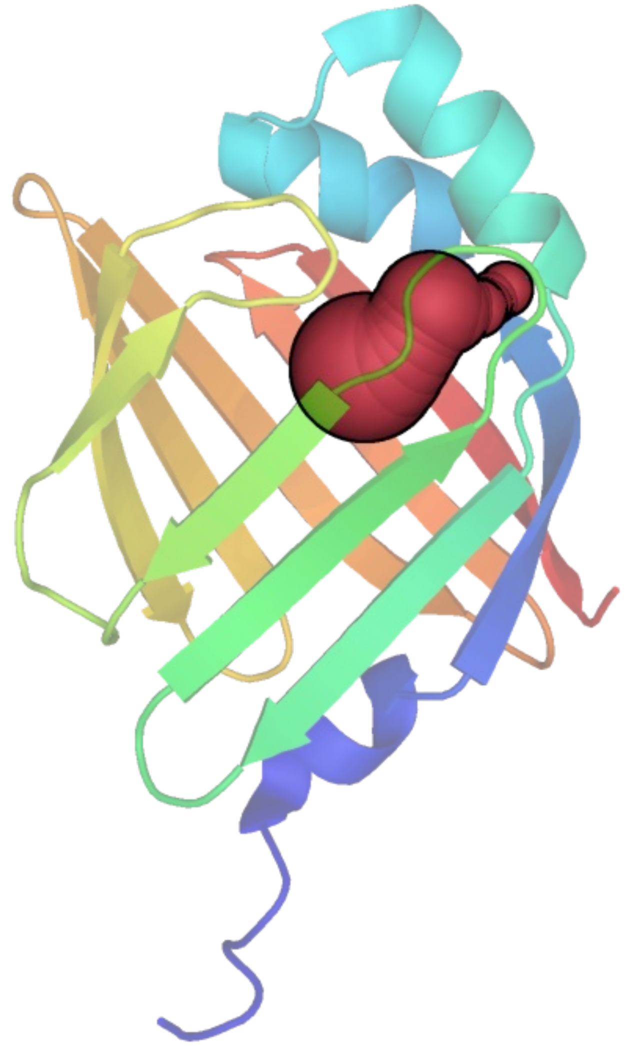

h

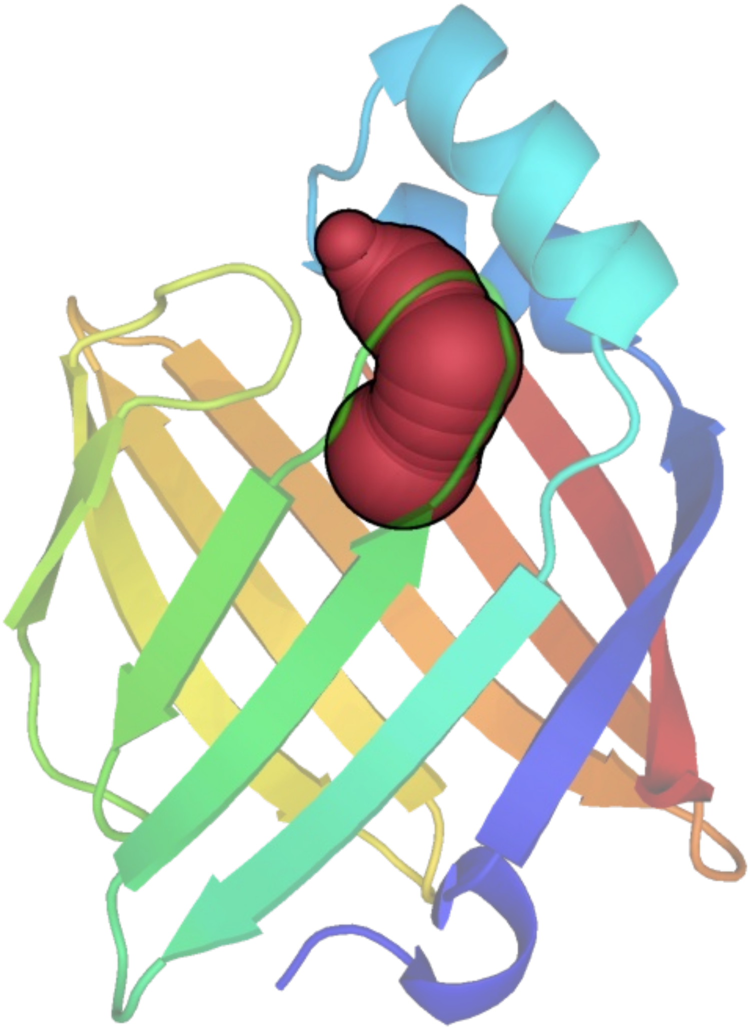

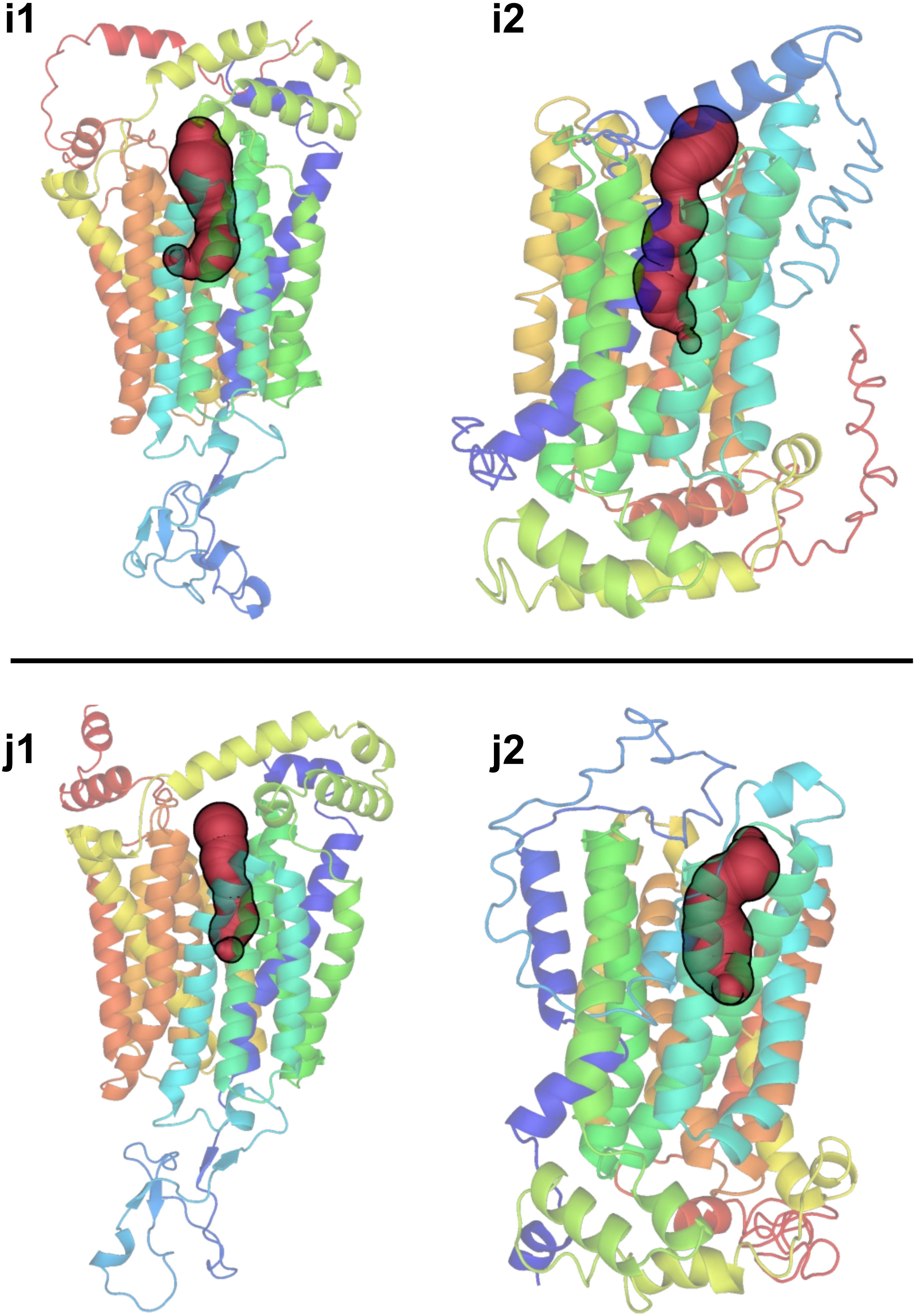
i1
i2
j1
j2

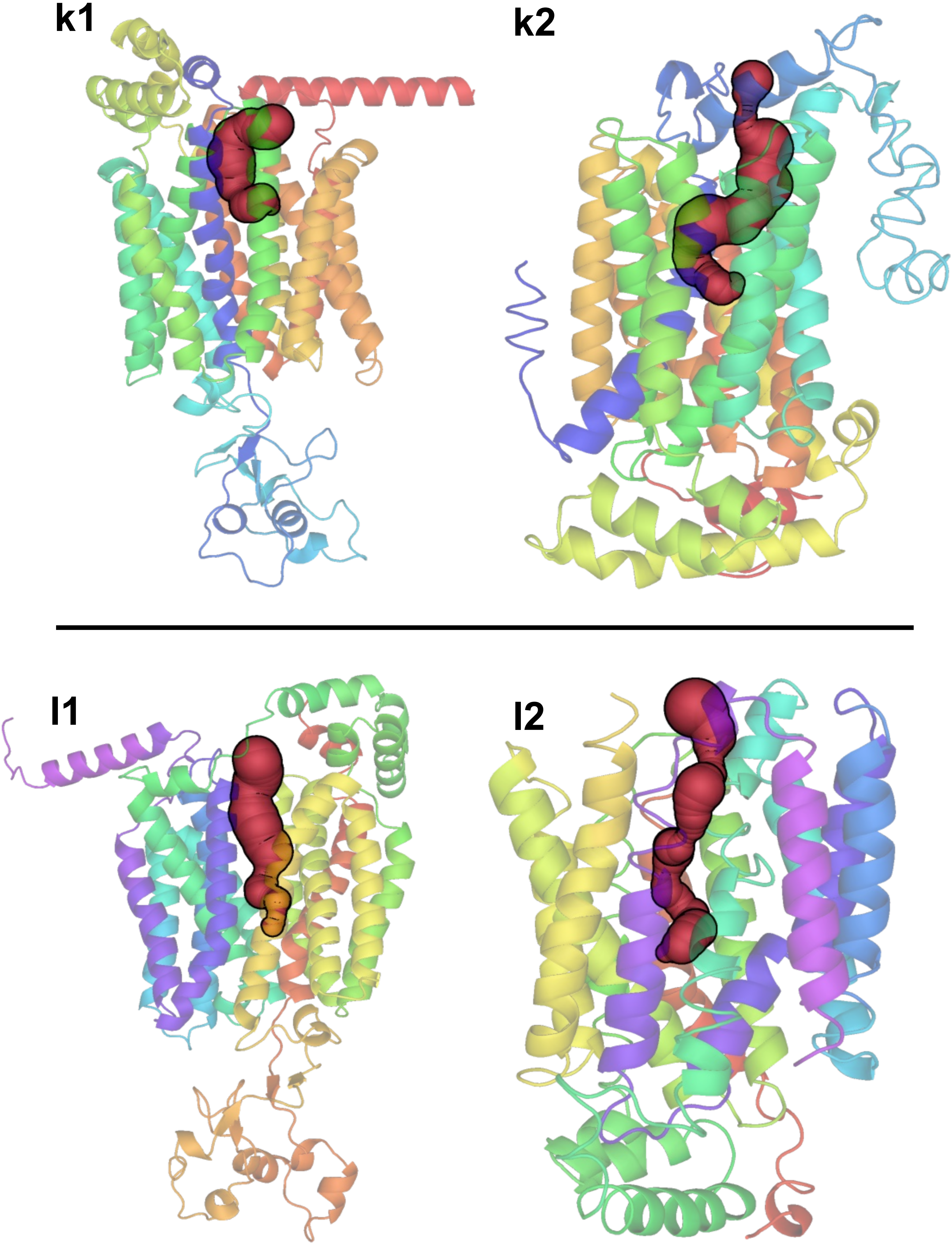
k1
k2
l1
l2

Figure S1. Visual representation of the studied set of proteins. The pockets reported are represented as red contours. **a.** Human Serum Albumin (HSA). Nine different pockets were considered for our docking studies, four of them for molecular dynamics simulations; **b.** Transthyretin (TTR) bound with PFOA (in both pockets). The PDB file contained the biological assembly, but the crystallographic structure was resolved only for the dimer (rainbow-coloured). The grey half of the protein was obtained, by Zhang *et al*. (2016), using symmetry; **c.** Thyroxine Binding Globulin (TBG); **d.** Fatty Acids Binding Protein (FABP), Liver isoform; **e.** FABP, Intestinal isoform; **f.** FABP, heart isoform; **g.** FABP, adipocyte isoform; **h.** Peripheral myeling Protein 2 (PmP2) isoform; **i.**Organic Anion Transporters; the “inward” isoforms are reported on the left, the “outward” isoforms on the right. **i1** and **i2**: OAT1; **j1** and **j2:** OAT3; **k1** and **k2:** OAT4; **l1** and **l2:** URAT1.

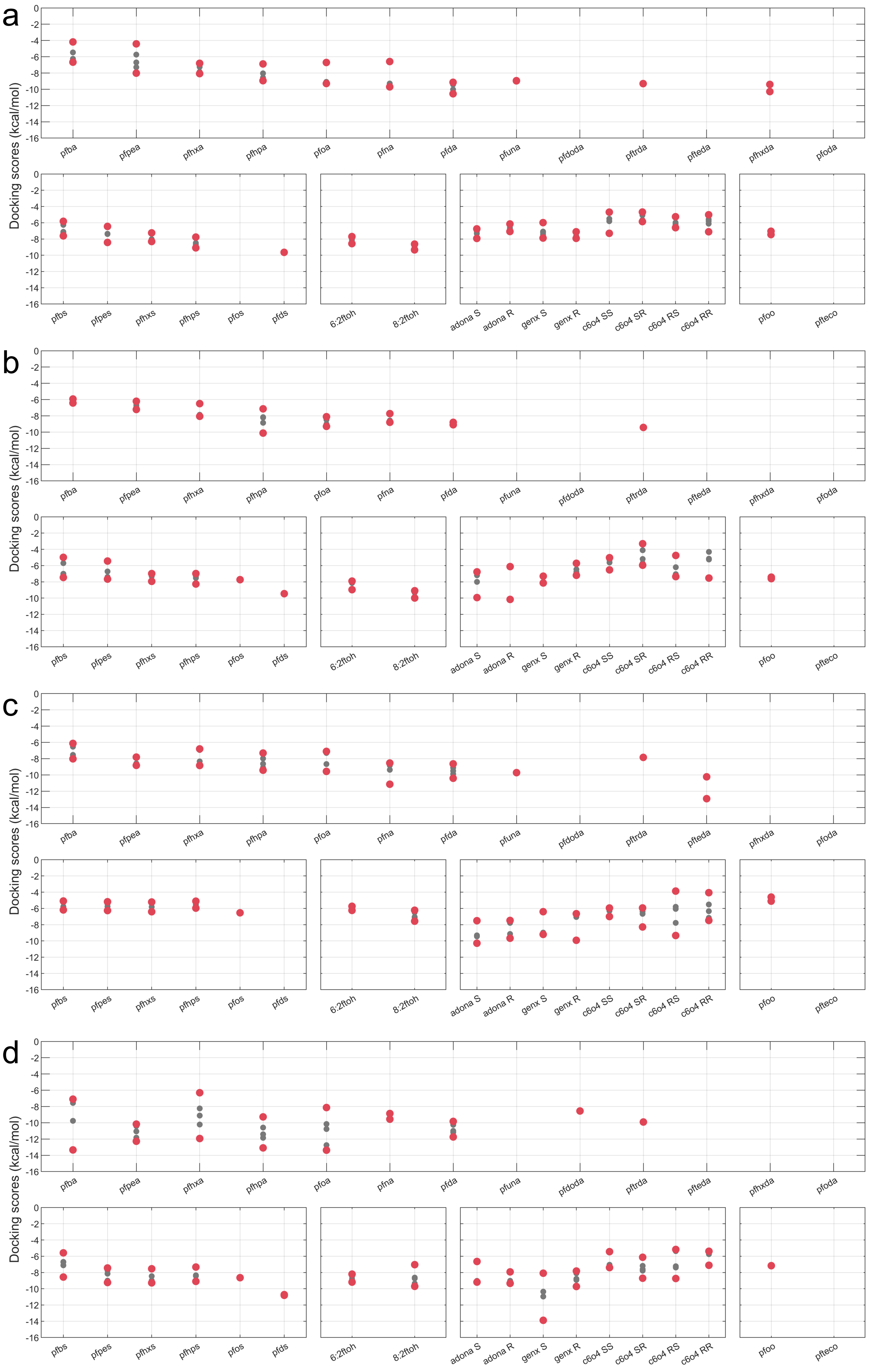
a
b
c
d
Docking scores (kcal/mol)
0
-2
-4
-6
-8
-10
-12
-14
-16
pfba
pfpea
pfhxa
pfhpa
pfoa
pfna
pfda
pfuna
pfdoda
pftrda
pfteda
pfhxda
pfoda
pfbs
pfpes
pfhxs
pfhps
pfos
pfds
6:2ftoh
8:2ftoh
adona S
adona R
genx S
genx R
c6o4 SS
c6o4 SR
c6o4 RS
c6o4 RR
pfoo
pfteco

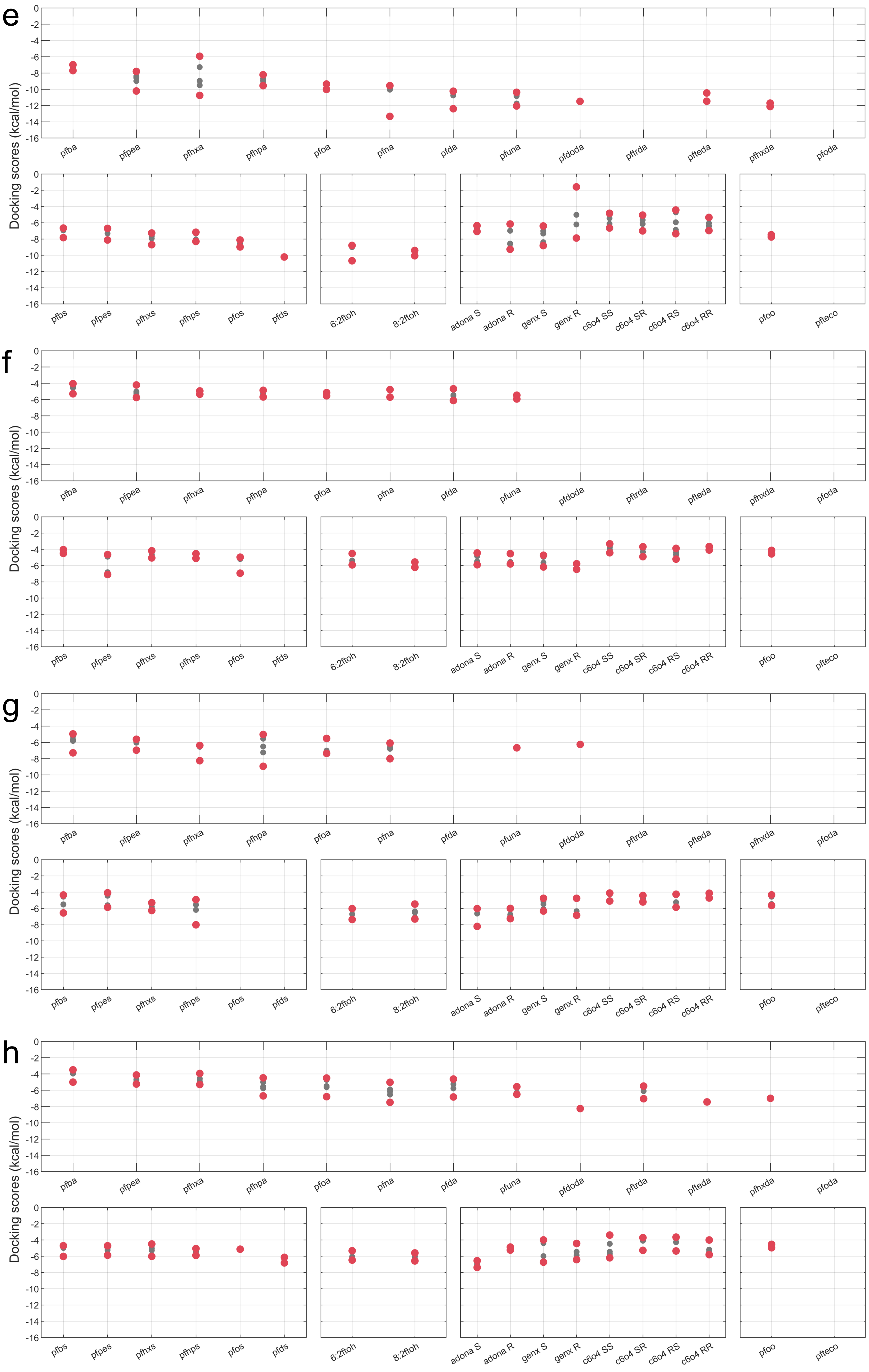
e
f
g
h
Docking scores (kcal/mol)
0
-2
-4
-6
-8
-10
-12
-14
-16
pfba
pfpea
pfhxa
pfhpa
pfoa
pfna
pfda
pfuna
pfdoda
pftrda
pfteda
pfhxda
pfoda
pfbs
pfpes
pfhxs
pfhps
pfos
pfds
6:2ftoh
8:2ftoh
adona S
adona R
genx S
genx R
c6o4 SS
c6o4 SR
c6o4 RS
c6o4 RR
pfoo
pfteco

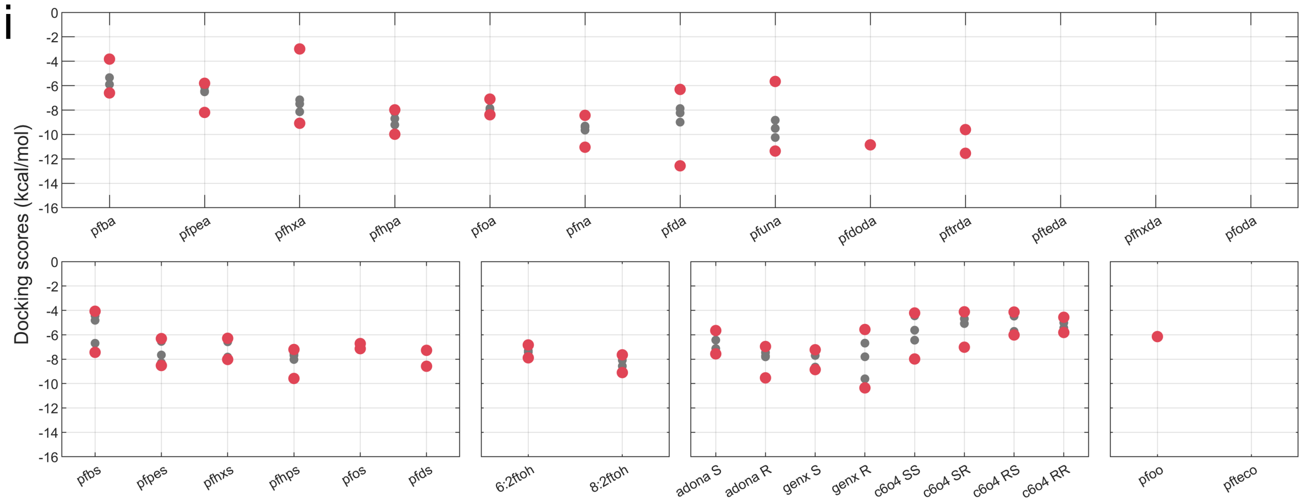

Figure S2. Docking scores (kcal/mol) for PFAS series docked in the different pockets of HSA. Graphs are divided by compound classes, maxima and minima of each PFAS are reported in red dots, while intermediate poses are reported in grey. **a.** Fatty Acid 1 pocket (FA1); **b.** Fatty Acid 2 pocket (FA2); **c.** Fatty Acid 3 pocket (FA3 or Sudlow II); **d.** Fatty Acid 4 pocket (FA4 or Sudlow II); **e.** Fatty Acid 5 pocket (FA5); **f.** Fatty Acid 6 pocket (FA6); **g.** Fatty Acid 7 (FA7 or Sudlow I); **h.** Cleft; **i.** Lowcleft.

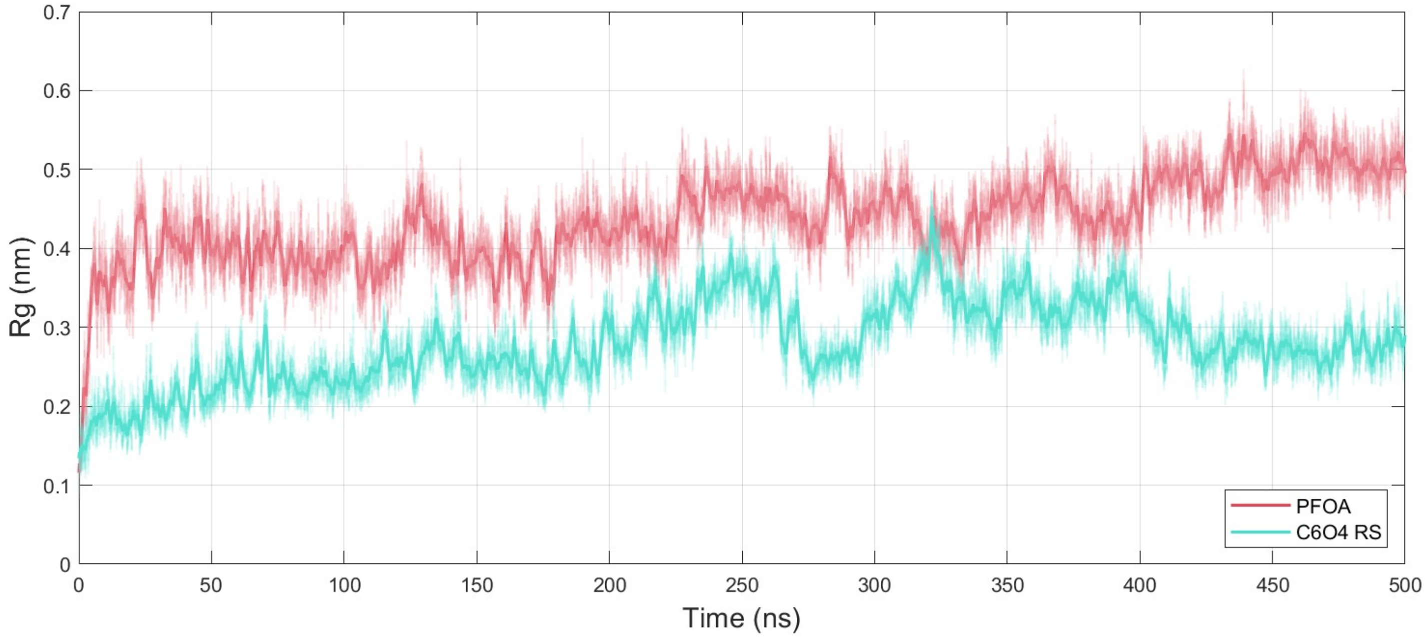

Figure S3. RMSD (nm) for the MD simulations of HSA complexed with PFOA and cC6O4 RS. The RMSD was calculated through GROMACS 4.6.1 package with the starting frame as reference structure. The opaque line is the unweighted moving average, or rolling mean, calculated for 100 frames.

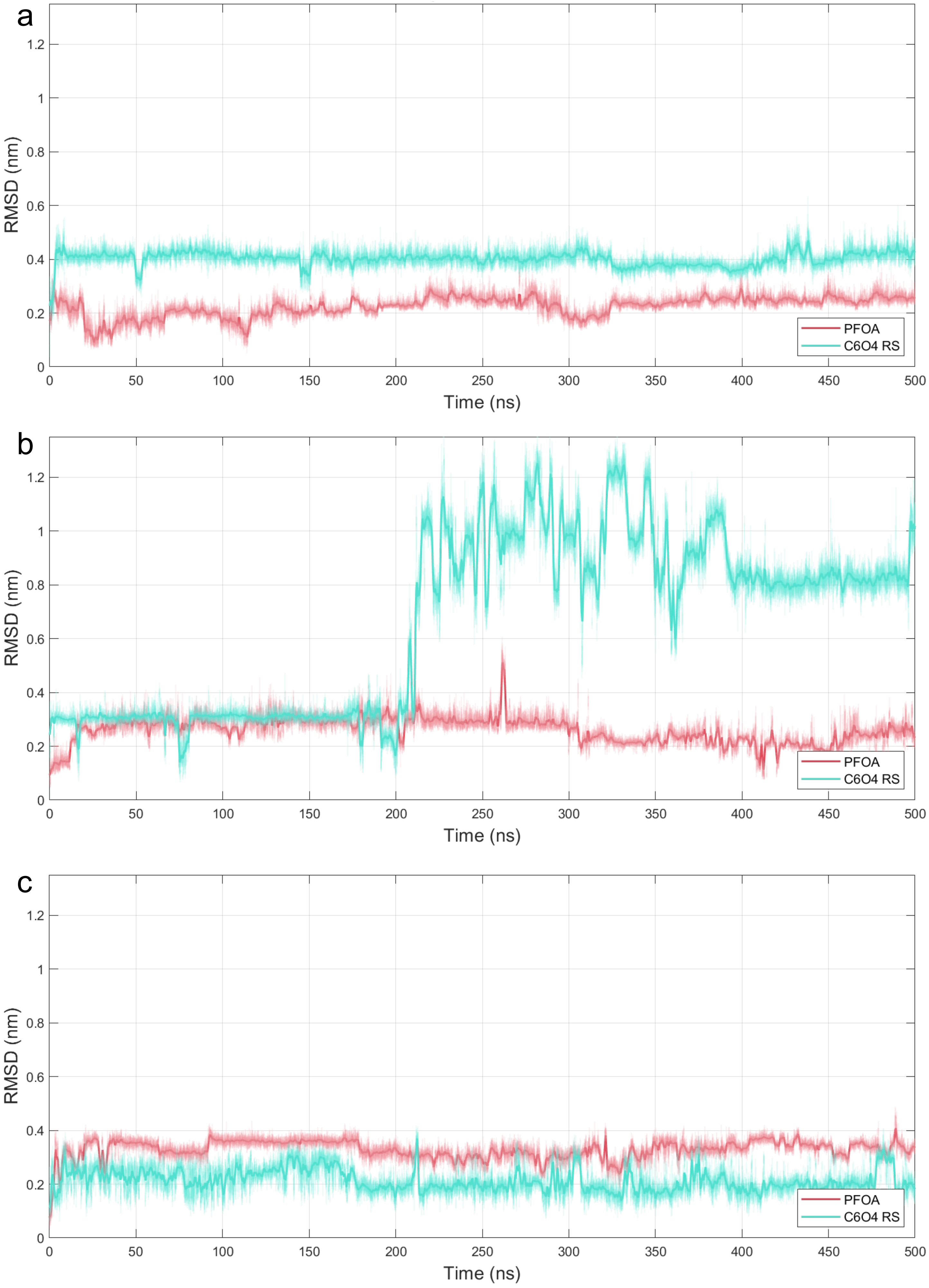
a
b
c
RMSD (nm)
Time (ns)
PFOA
C6O4 RS
0
0.2
0.4
0.6
0.8
1
1.2
0
50
100
150
200
250
300
350
400
450
500

d

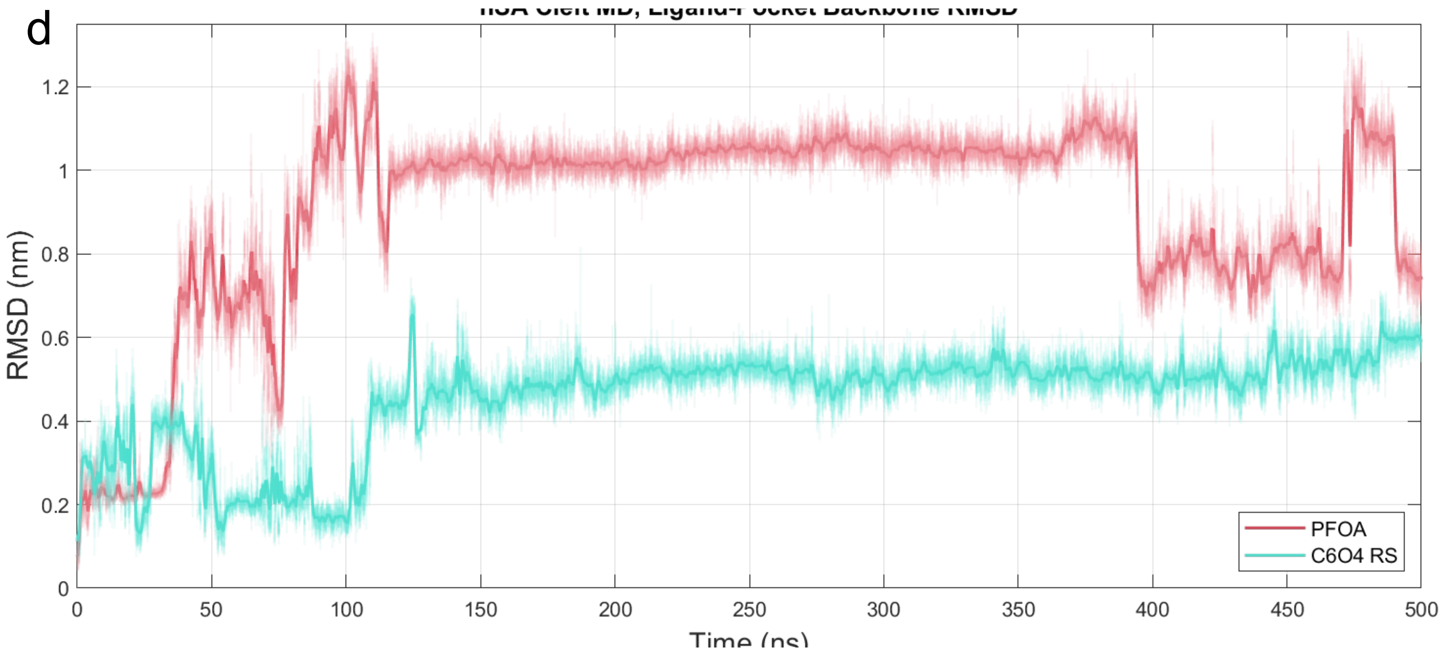

Figure S4. RMSD (nm) calculated for PFOA (in red) and cC6O4 (in turquoise) in complex with HSA. Least square fitting was performed on the pocket residues with the starting frame as reference structure, and the rmsd was then calculated (through GROMACS 4.6.1 package). The opaque line is the unweighted moving average, or rolling mean, calculated for 100 frames. **a.** FA4 pocket; **b.** FA6 pocket; **c.** FA7 pocket; **d.** Cleft.

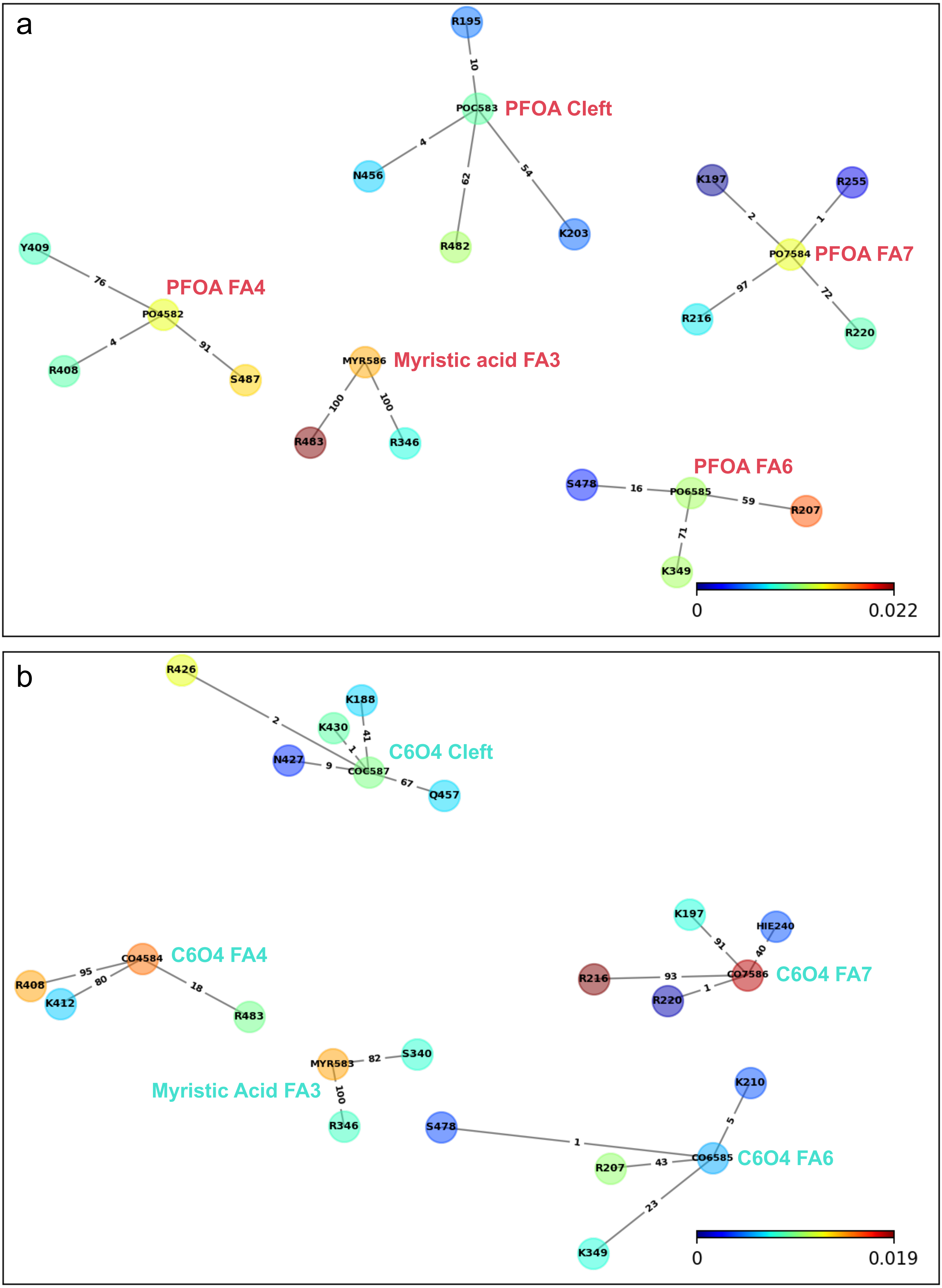

Figure S5. Hbond persistency calculated for the MD simulations of HSA complexed with PFOA (**a**) and cC6O4 (**b**). Each dot (node) represents a residue, in its one-letter code and number, or a ligand. Their color represents the number of link incidents on that node according to degree centrality. The number on each link represents the occupancy of each H-bond interaction expressed as a percentage of simulation time. Occupancies lower than 1% are not reported. Data have been calculated with the Bridge2 package.

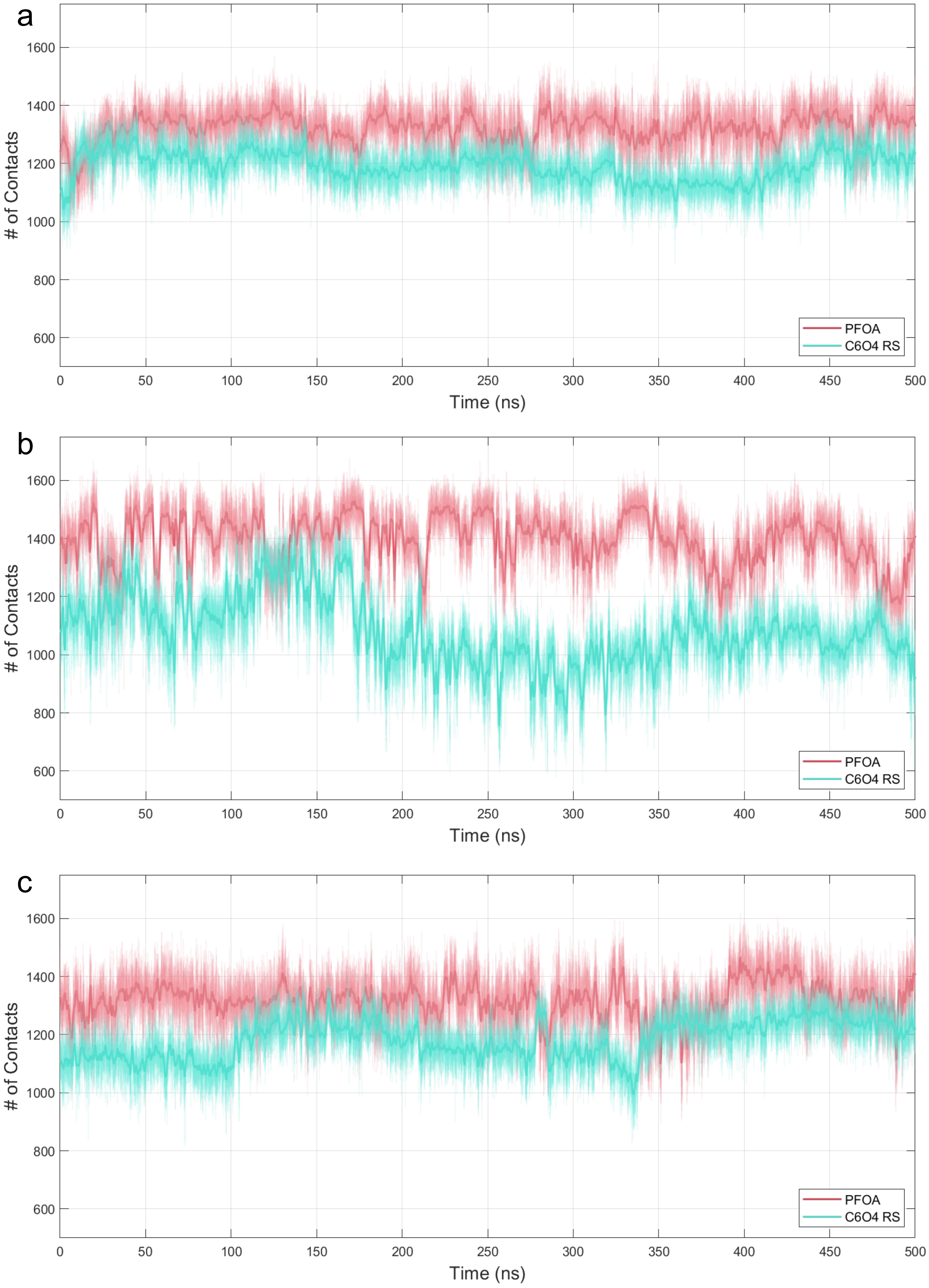
a
b
c
# of Contacts
Time (ns)
PFOA
C6O4 RS
1600
1400
1200
1000
800
600
0
50
100
150
200
250
300
350
400
450
500

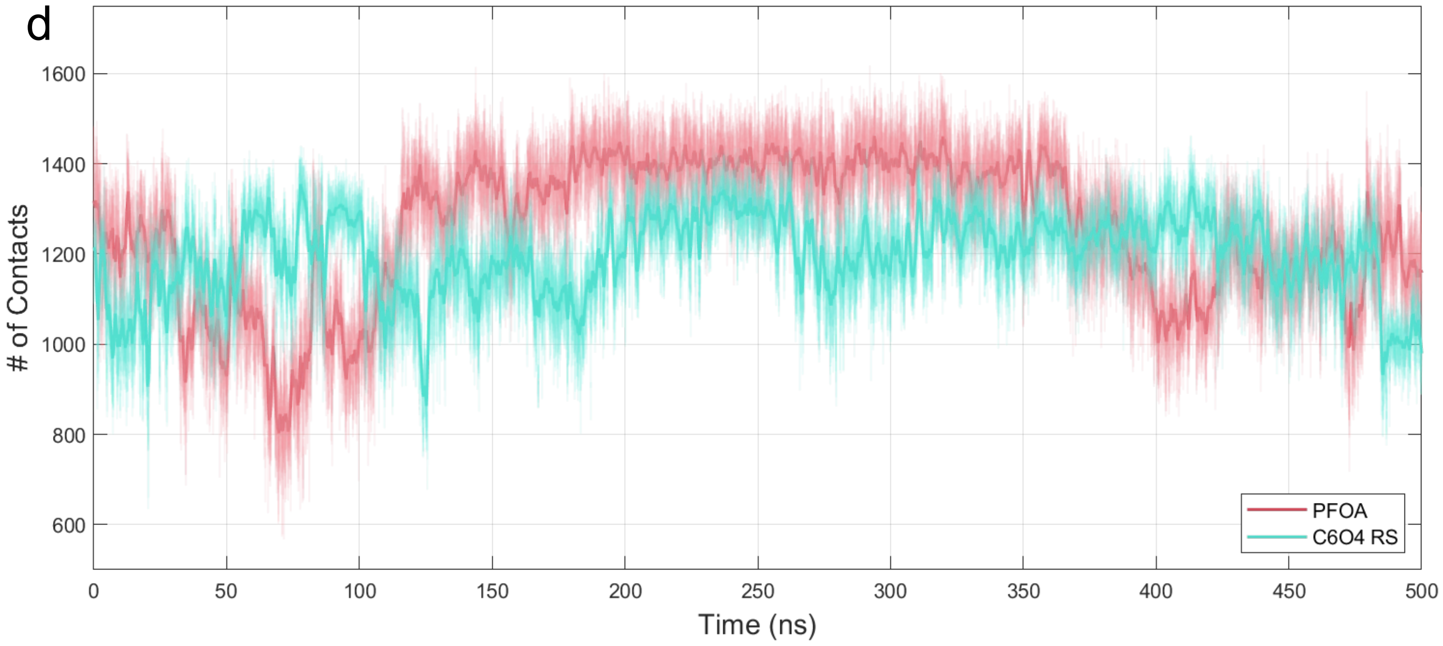

Figure S6. Number of contacts calculated for PFOA (in [coral] red) and cC6O4 (in turquoise) in complex with HSA. Any distance lower than 0.6 nms with any atom of the polymer was reported for the both ligands, and was calculated using GROMACS 4.6.1. **a.** FA4 pocket; **b.** FA6 pocket; **c.** FA7 pocket. **D.** Cleft.

compound	% bound to BSA [a)]	% recovery
cC6O4	62 ±3	99
PFOA	82 ±2	85

Table S2: binding of the compounds to bovine serum albumin (BSA) and recovery in dialysis equilibrium experiments

a) Data are presented as means ± SEM (C6O4, n = 7; PFOA, n=10). Statistical analyses were made using Student′s t-test for unpaired data: *** $p < 0.0001$ C6O4 vs. PFOA.

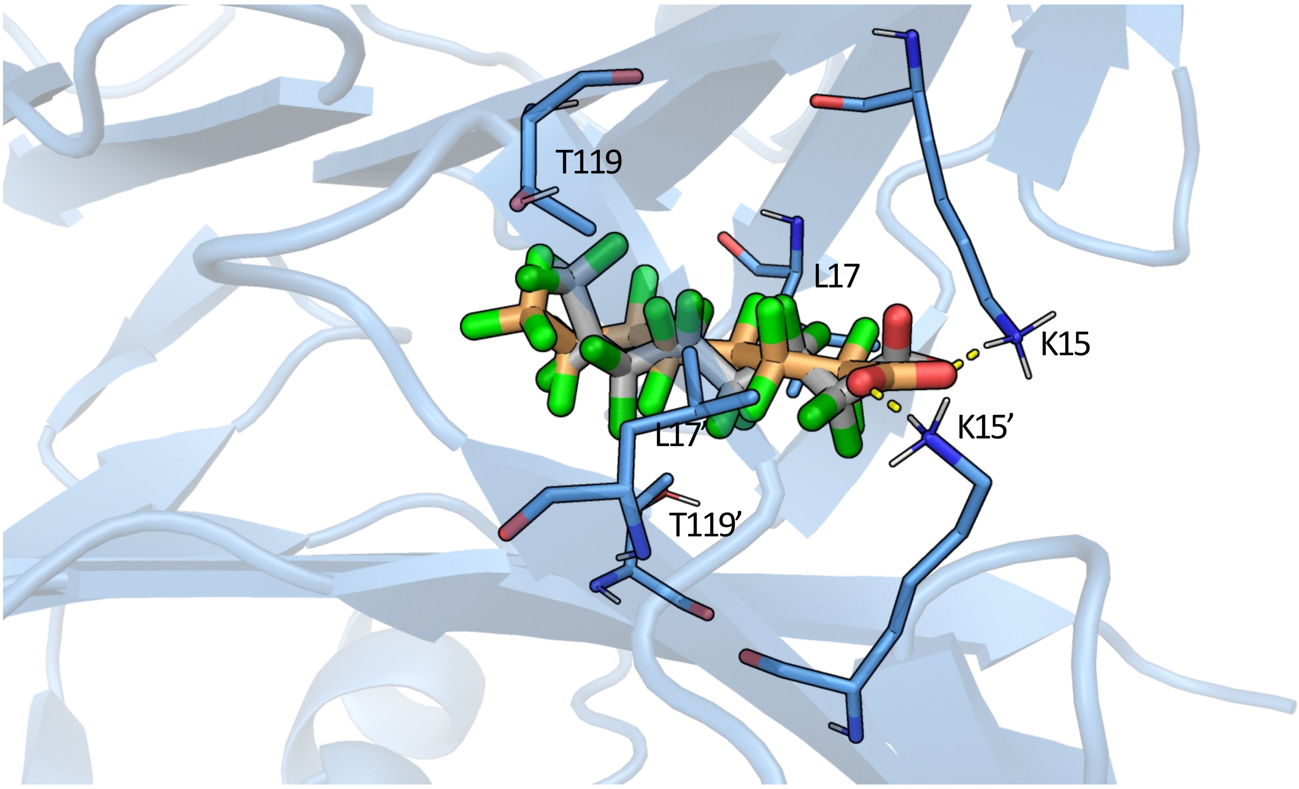

Figure S7. Superposition of the crystallographic pose (gray; PDB ID 5JID) and the docked pose (orange) of PFOA in TTR binding site after IFD. The protein is shown as cartoon, the ligands in capped sticks and the H-bonds as yellow dashed lines.

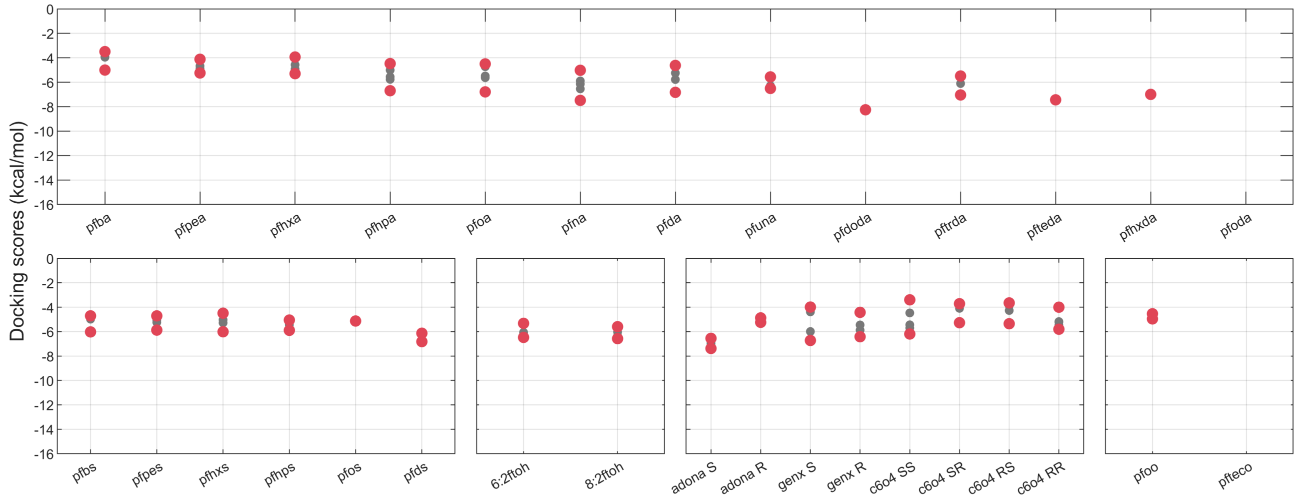

Figure S8. Docking scores (kcal/mol) for PFAS series docked in TTR. Graphs are divided by compound classes, maxima and minima of each PFAS are reported in red dots, while intermediate poses are reported in gray.

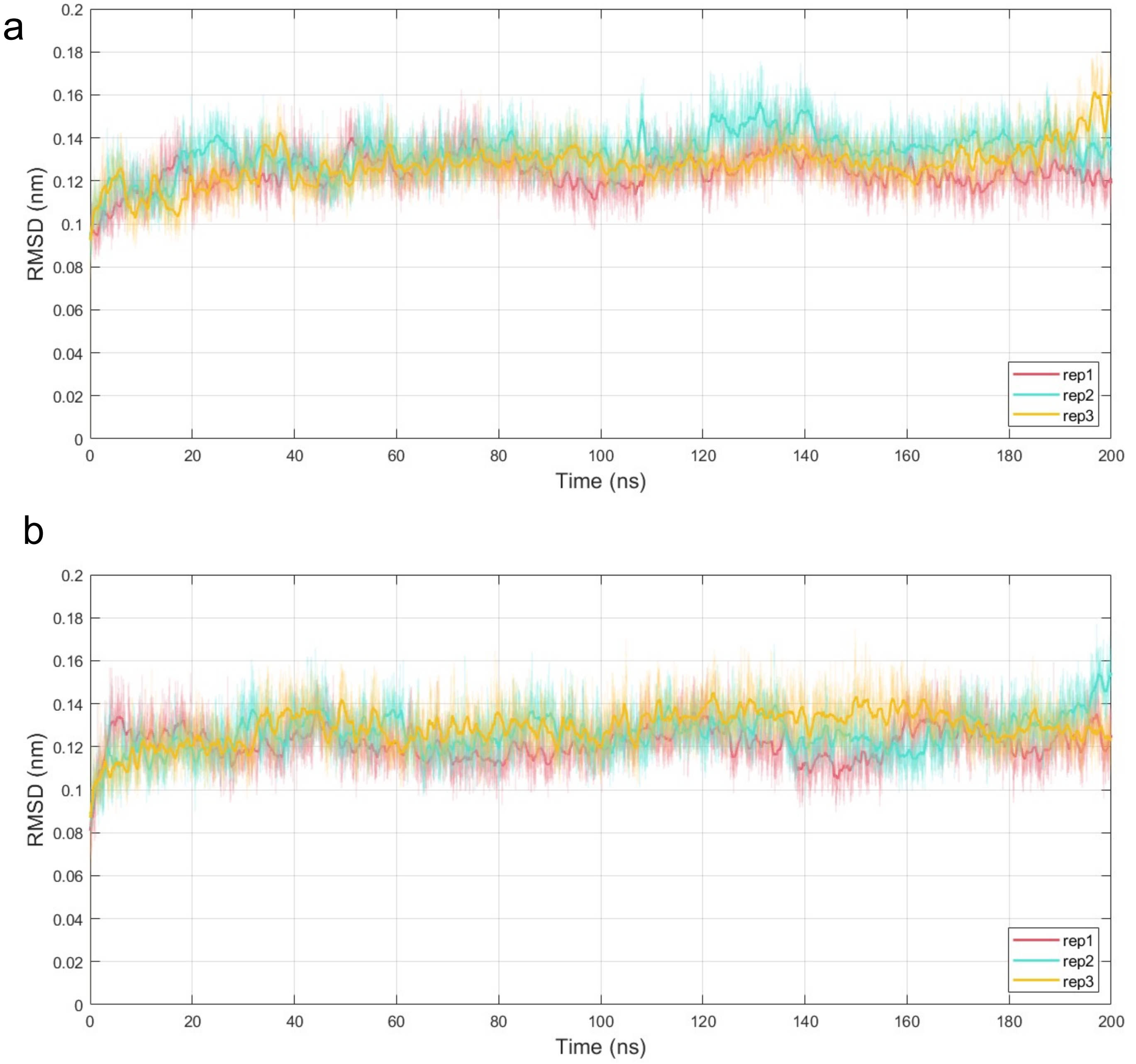

Figure S9. RMSD (nm) for the MD simulations of TTR complexed with PFOA (**a**) and cC6O4 RS (**b**). Calculations were performed with GROMACS 4.6.1 package on the protein backbone, using the starting frame as reference. The opaque line is the unweighted moving average, or rolling mean, calculated for 100 frames. The three lines correspond to the RMSD calculated for each MD replica.

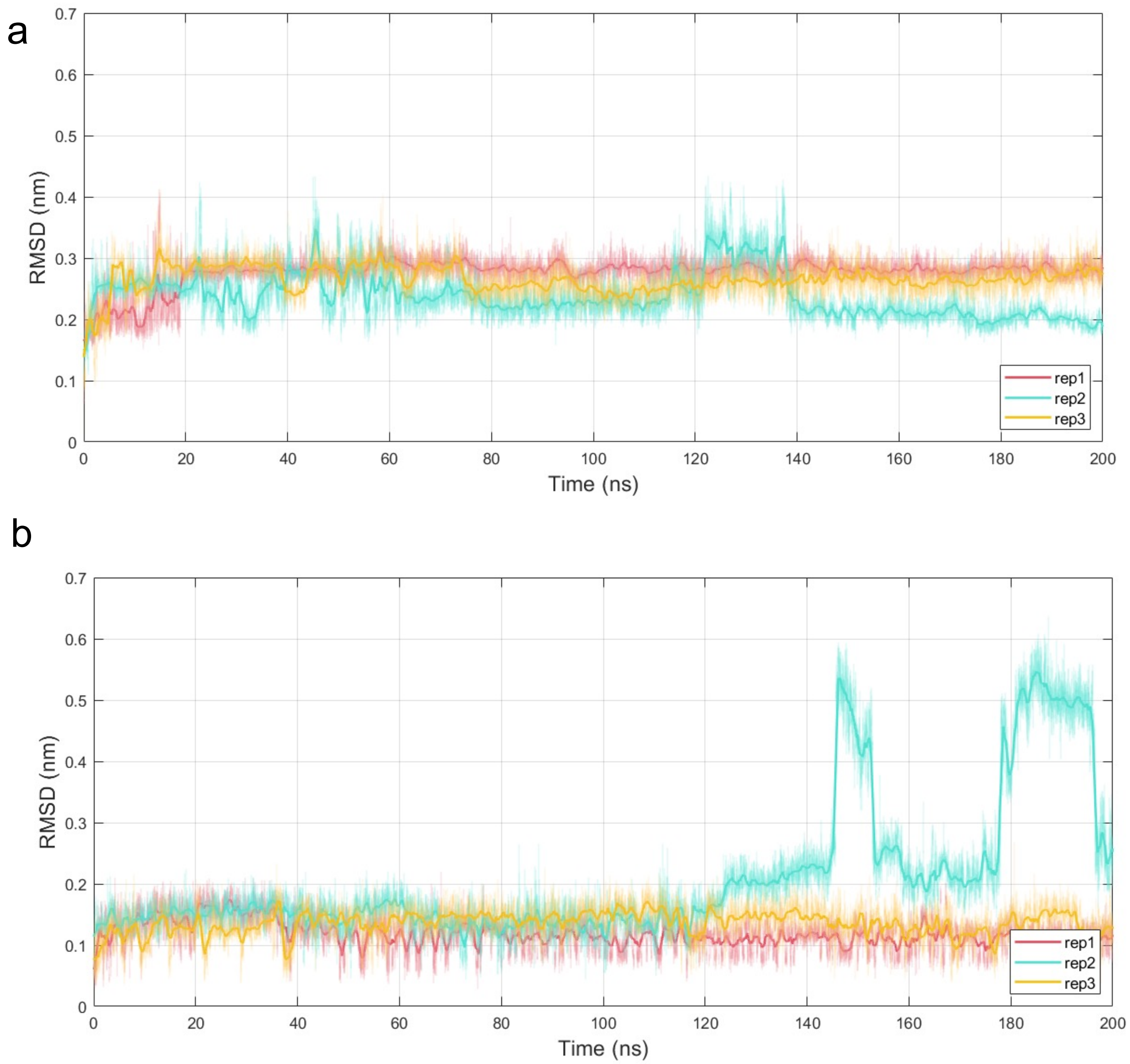

Figure S10. RMSD (nm) calculated for PFOA (a) and cC6O4 (b) in complex with TTR. Least square fitting was performed on the pocket residues with the starting frame as reference structure, and the rmsd was then calculated (through GROMACS 4.6.1 package). The opaque line is the unweighted moving average, or rolling mean, calculated for 100 frames. The three lines correspond to the RMSD calculated for each MD replica.

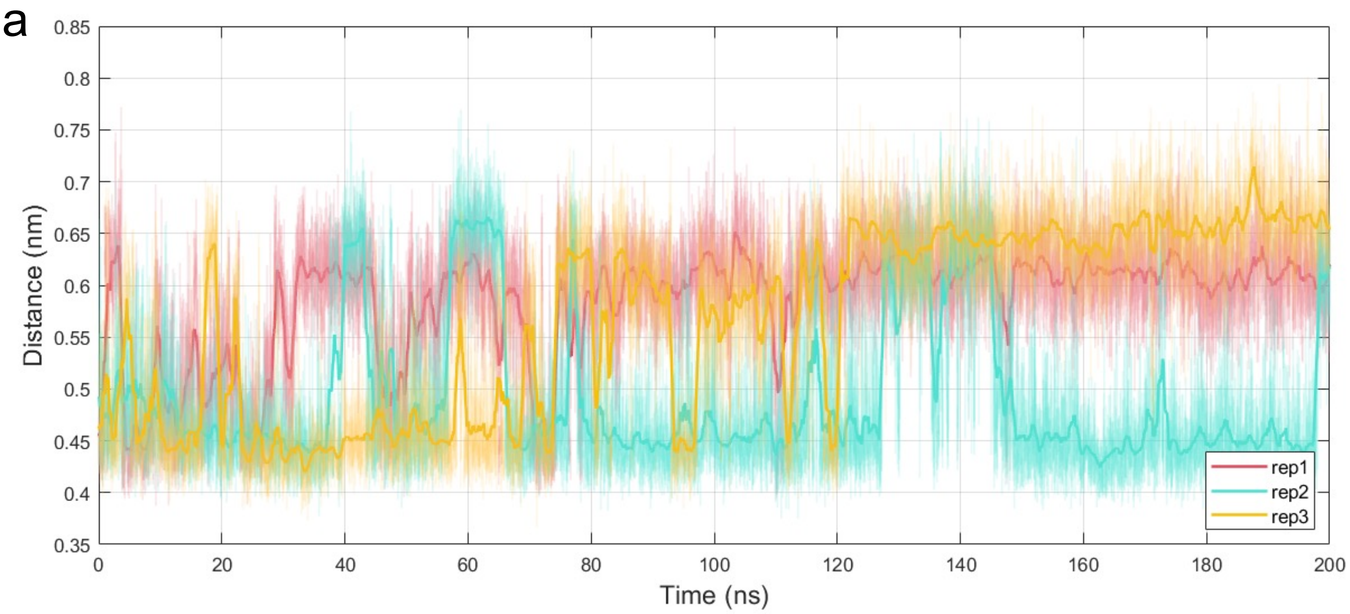

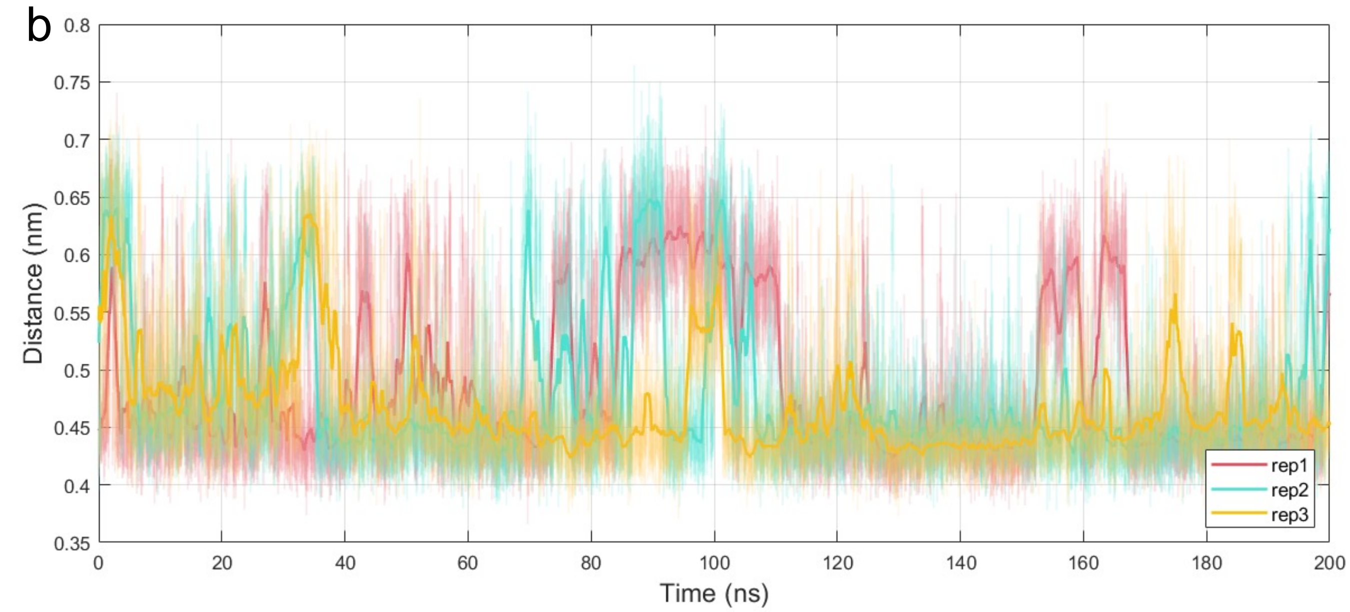

Figure S11. Distance (nm) between Thr119 and T4 in TTR:PFOA(**a**) and TTR:cC6O4 (**b**) complexes. Distances are calculated during the whole simulation through GROMACS 4.6.1 package. The opaque line is the unweighted moving average, or rolling mean, calculated for 100 frames. The three lines correspond to the distances calculated for each MD replica.

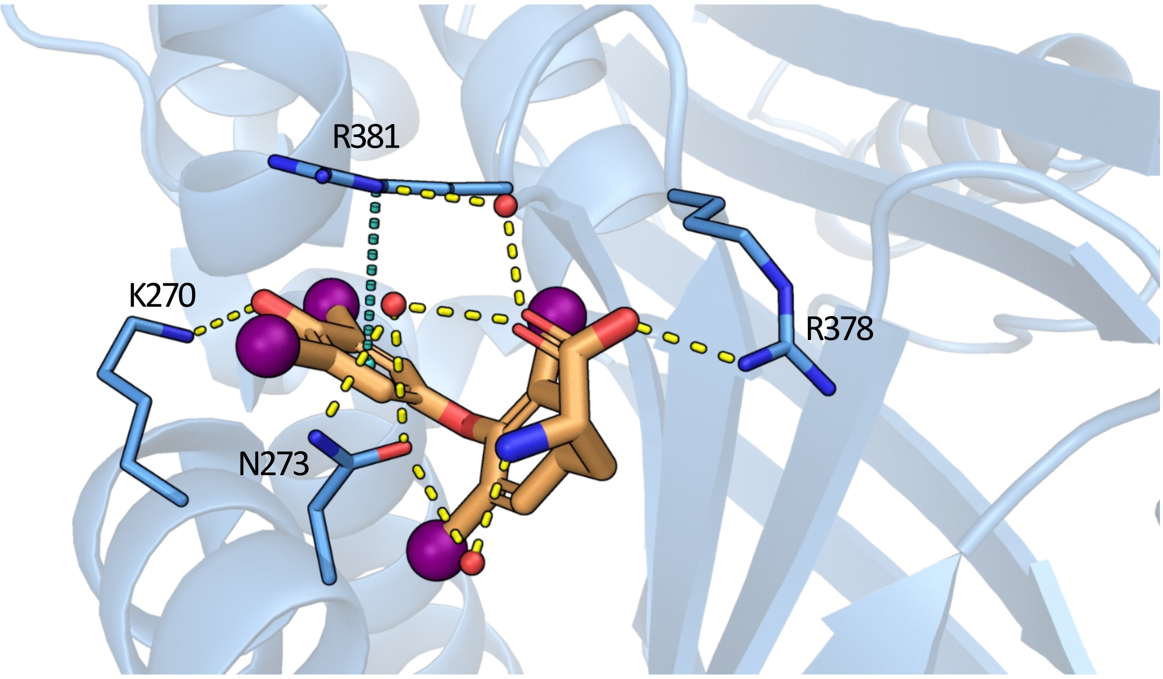

Figure S12. Crystallographic pose of T4 in TBG binding site (PDB ID 4X30). The protein is shown as cartoon, the ligands in capped sticks, the H-bonds as yellow dashed lines and the cation-π stacking as turquoise dashed line.

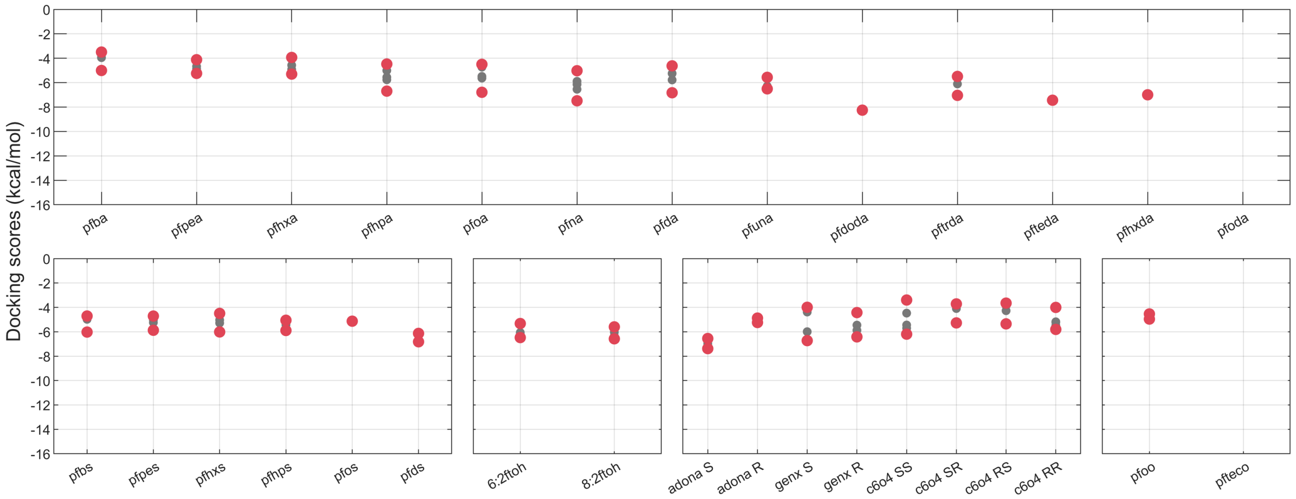

Figure S13. Docking scores (kcal/mol) for PFAS series docked in TBG. Graphs are divided by compound classes, maxima and minima of each PFAS are reported in red dots, while intermediate poses are reported in grey.

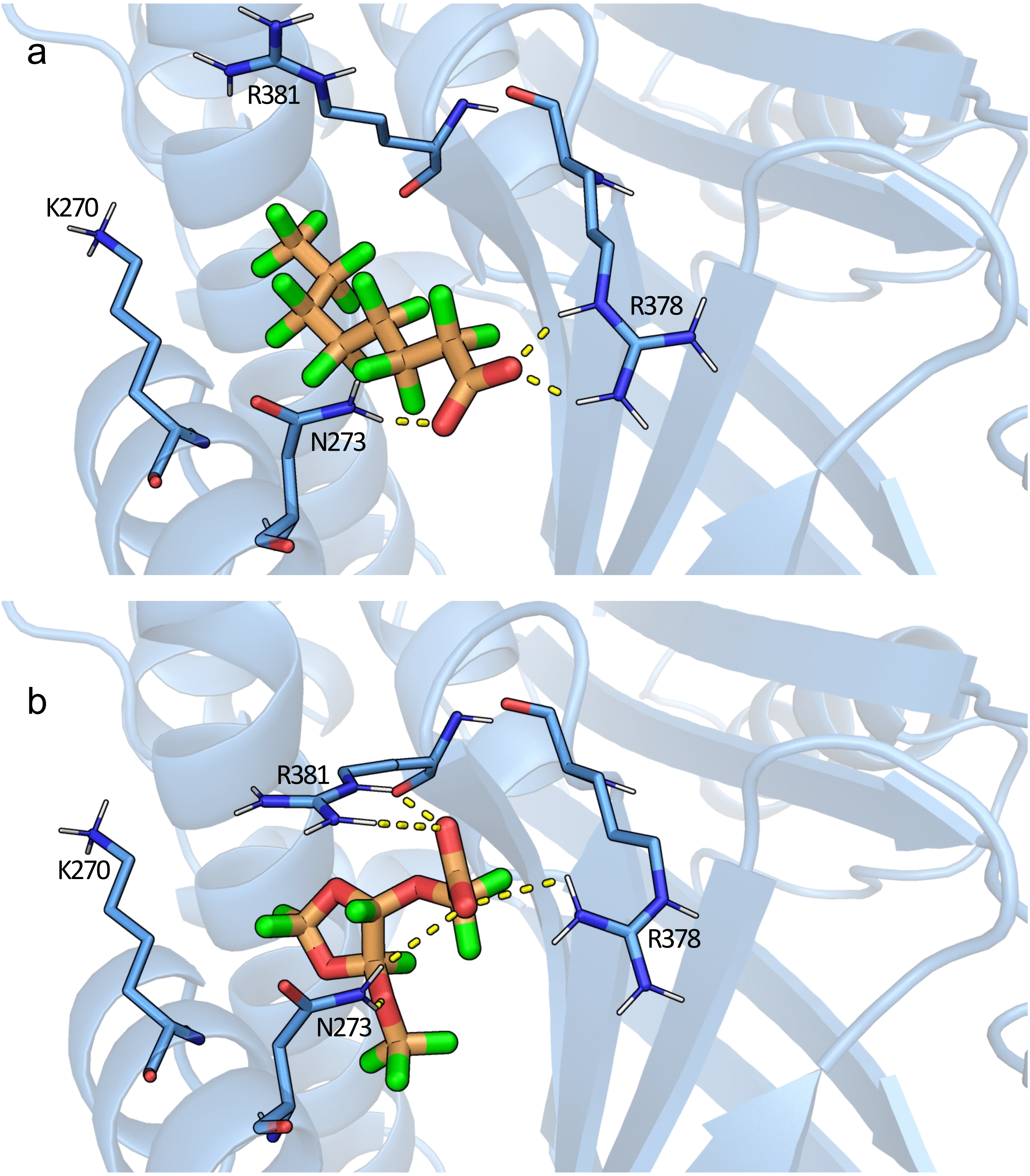

Figure S14. Docking pose of PFOA and cC6O4 in TBG. The protein is shown as cartoon, the ligands in capped sticks and the H-bonds as yellow dashed lines.

a

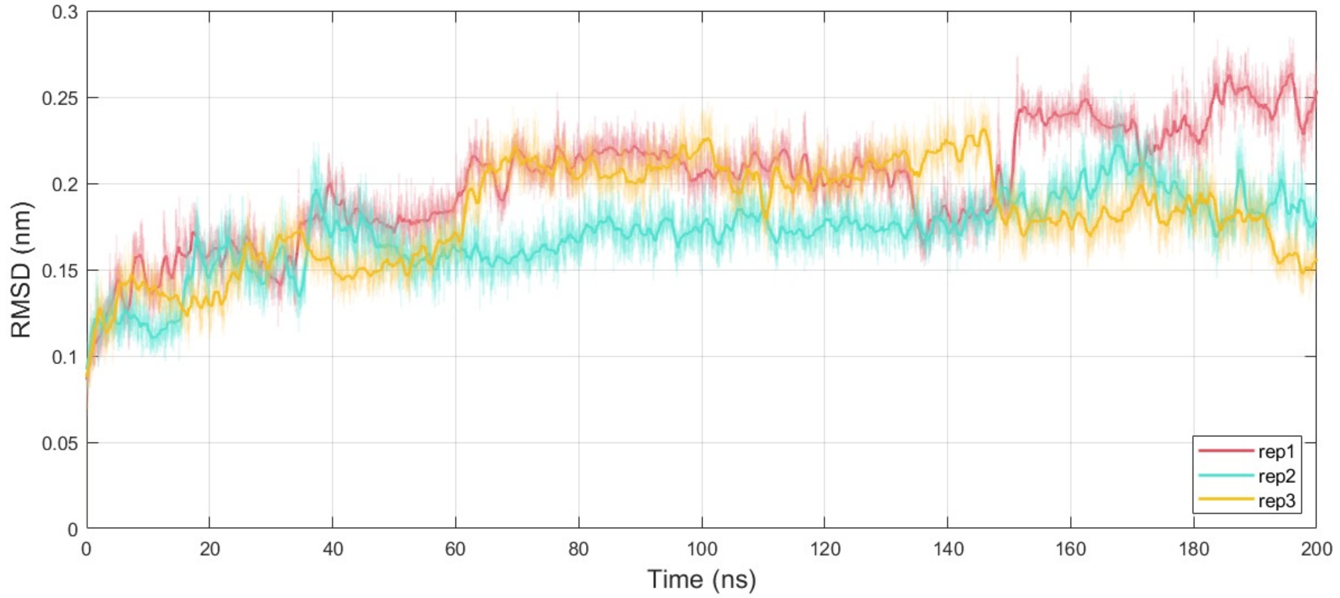

b

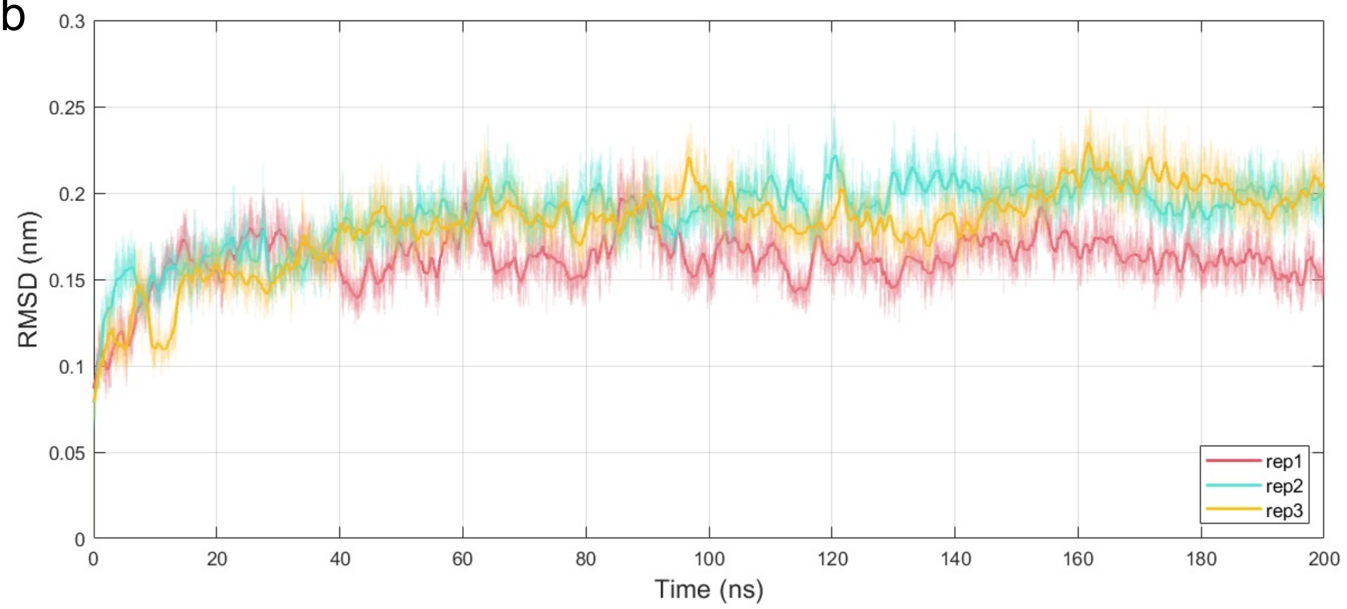

Figure S15. RMSD (nm) for the MD simulations of TBG complexed with PFOA (**a**) and cC6O4 (**b**). Calculations were performed with GROMACS 4.6.1 package on the protein backbone, using the starting frame as reference. The opaque line is the unweighted moving average, or rolling mean, calculated for 100 frames. The three lines correspond to the RMSD calculated for each MD replica.

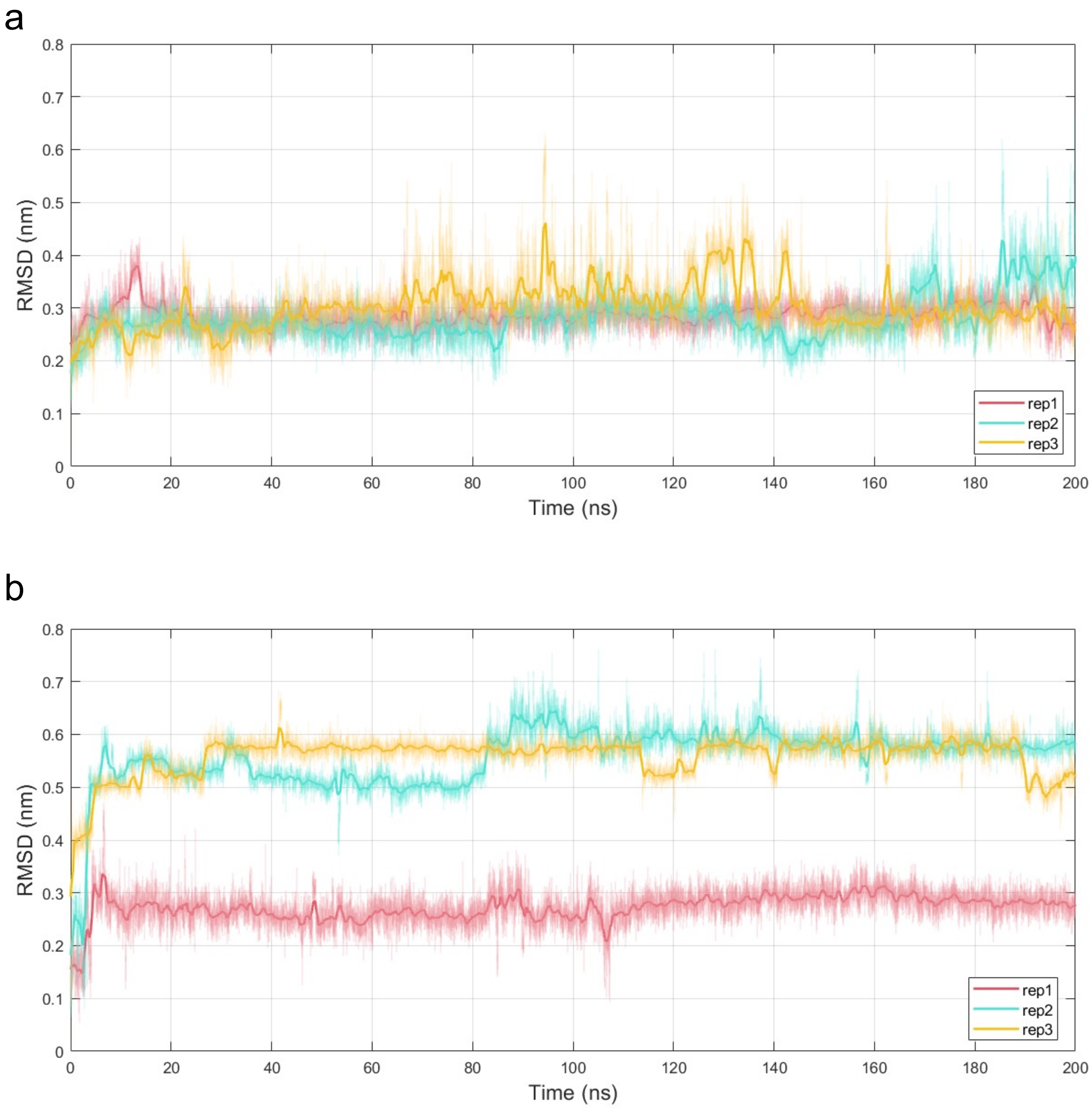

Figure S16. RMSD (nm) calculated for PFOA (**a**) and cC6O4 (**b**) in complex with TBG. Least square fitting was performed on the pocket residues with the starting frame as reference structure, and the rmsd was then calculated (through GROMACS 4.6.1 package). The opaque line is the unweighted moving average, or rolling mean, calculated for 100 frames. The three lines correspond to the RMSD calculated for each MD replica.

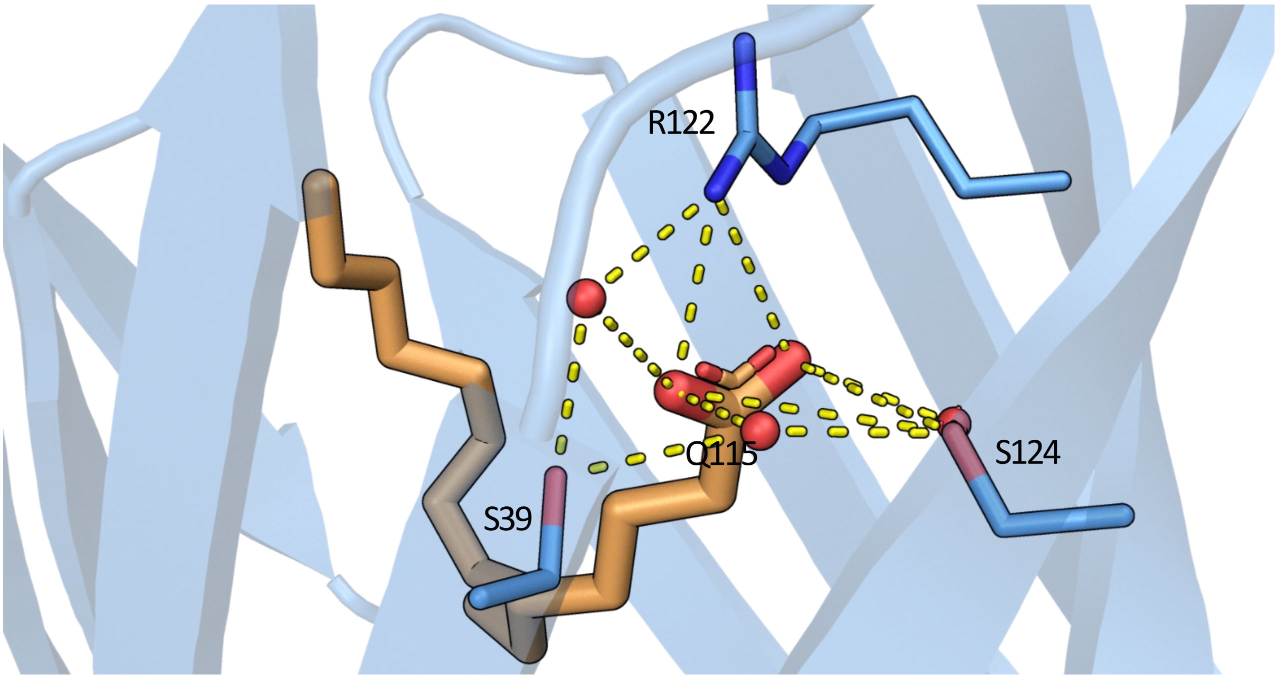

Figure S17. Crystallographic pose of palmitic acid in L-FABP binding site (PDB ID 3STK). The protein is shown as cartoon, the ligands in capped sticks and the H-bonds as yellow dashed lines.

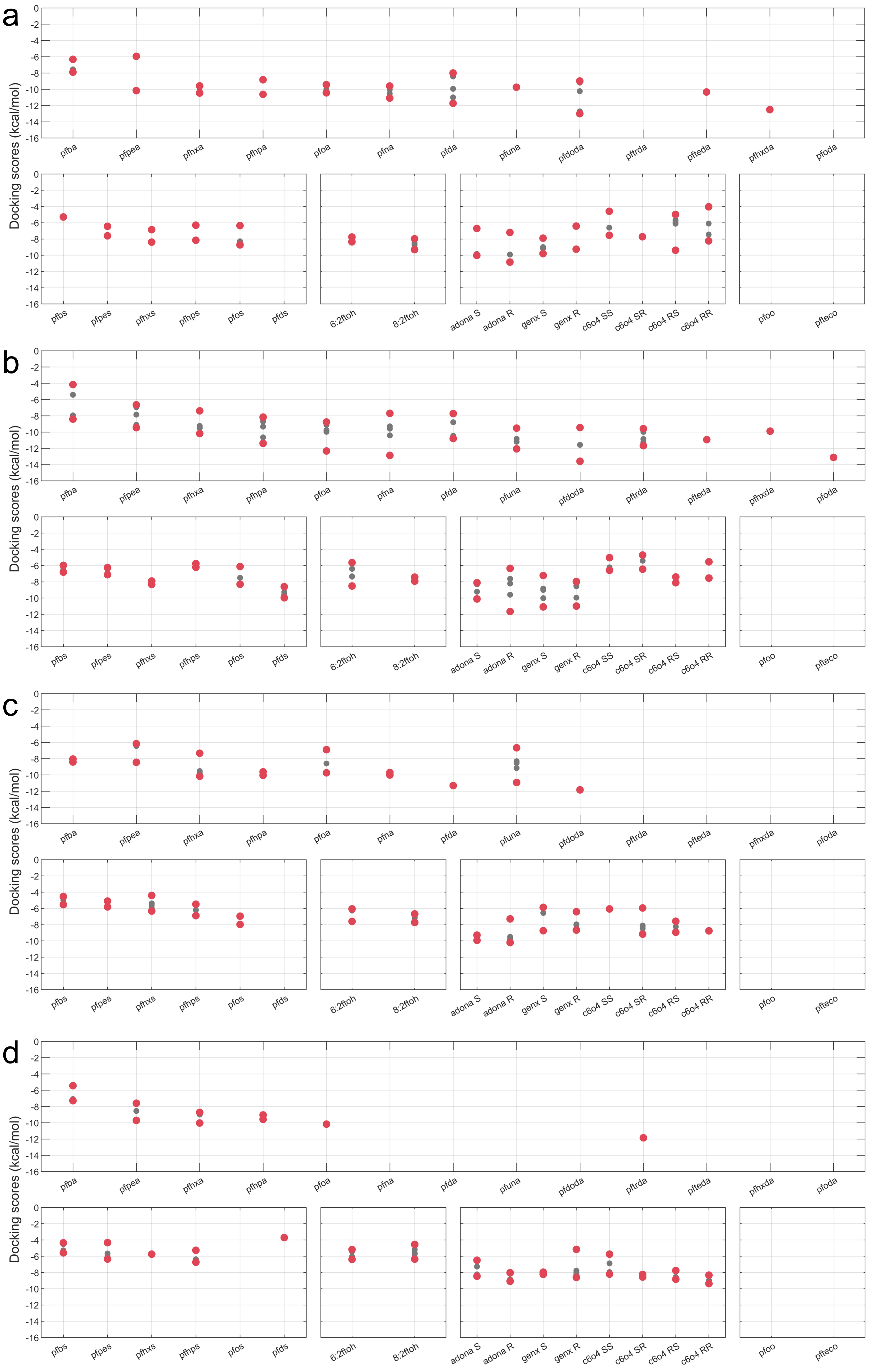
a
b
c
d
Docking scores (kcal/mol)
0
-2
-4
-6
-8
-10
-12
-14
-16
pfba
pfpea
pfhxa
pfhpa
pfoa
pfna
pfda
pfuna
pfdoda
pftrda
pfteda
pfhxda
pfoda
pfbs
pfpes
pfhxs
pfhps
pfos
pfds
6:2ftoh
8:2ftoh
adona S
adona R
genx S
genx R
c6o4 SS
c6o4 SR
c6o4 RS
c6o4 RR
pfoo
pfteco

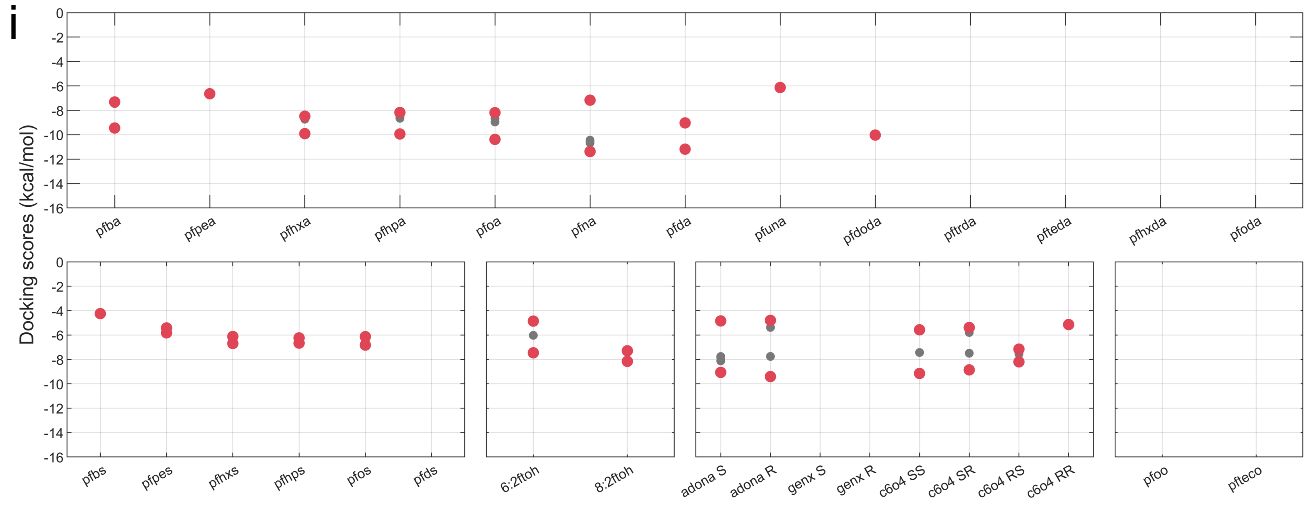

Figure S18. Docking scores (kcal/mol) for PFAS series docked in FABPs. Graphs are divided by compound classes, maxima and minima of each PFAS are reported in red dots, while intermediate poses are reported in gray. **a.** L-FABP; **b.** I-FABP; **c.** H-FABP; **d.** A-FABP; **e.** PmP2.

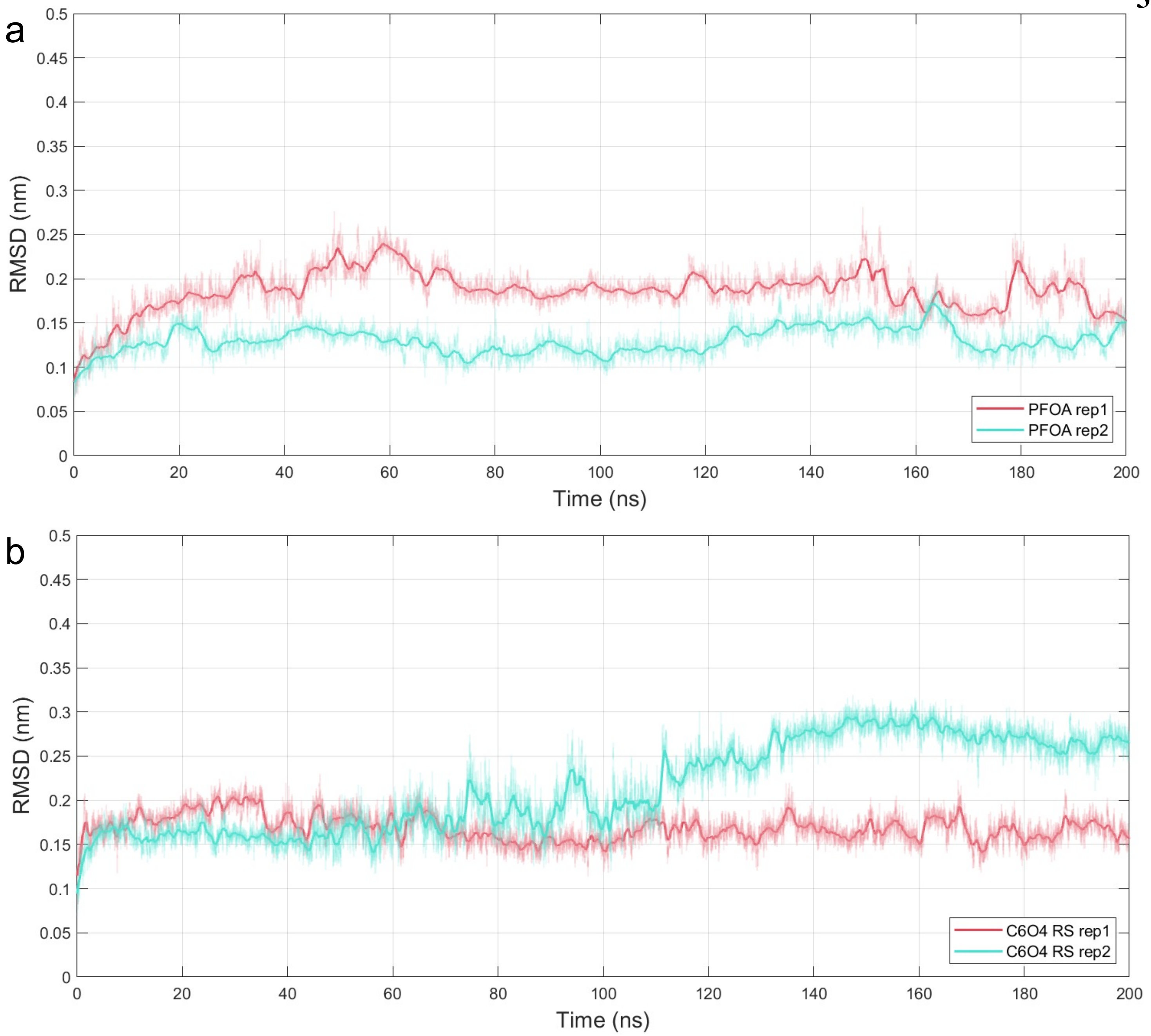

Figure S19. RMSD (nm) of the backbone for the MD simulations of L-FABP complexed with PFOA and cC6O4 RS. The RMSD was calculated through GROMACS 4.6.1 package with the starting frame as reference structure. The opaque line is the unweighted moving average, or rolling mean, calculated for 100 frames.

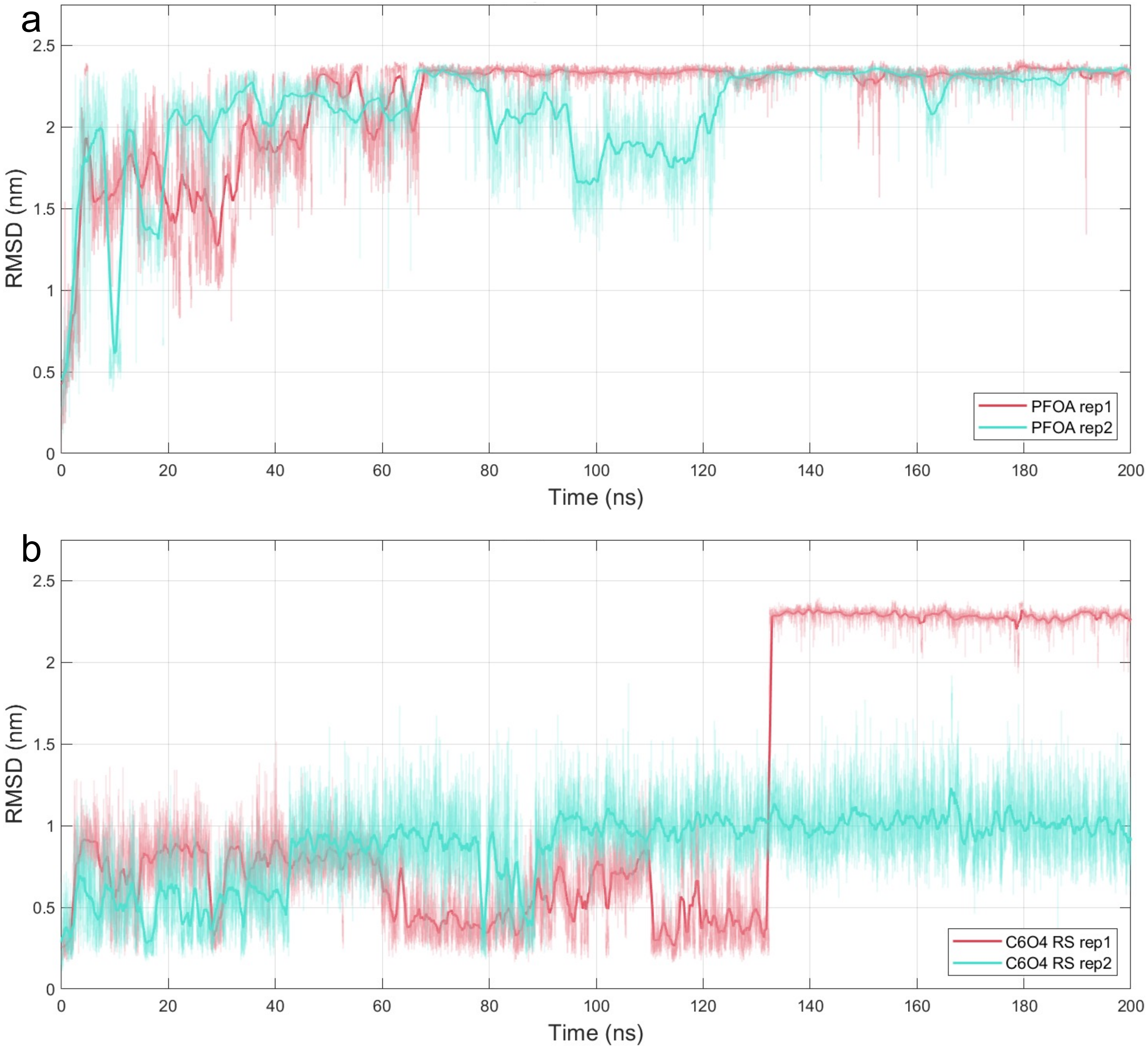

Figure S20. RMSD (nm) for the MD simulations of L-FABP complexed with PFOA (**a**) and cC6O4 (**b**). Least square fitting was performed on the pocket residues with the starting frame as reference structure, and the rmsd was then calculated (through GROMACS 4.6.1 package). The opaque line is the unweighted moving average, or rolling mean, calculated for 100 frames.

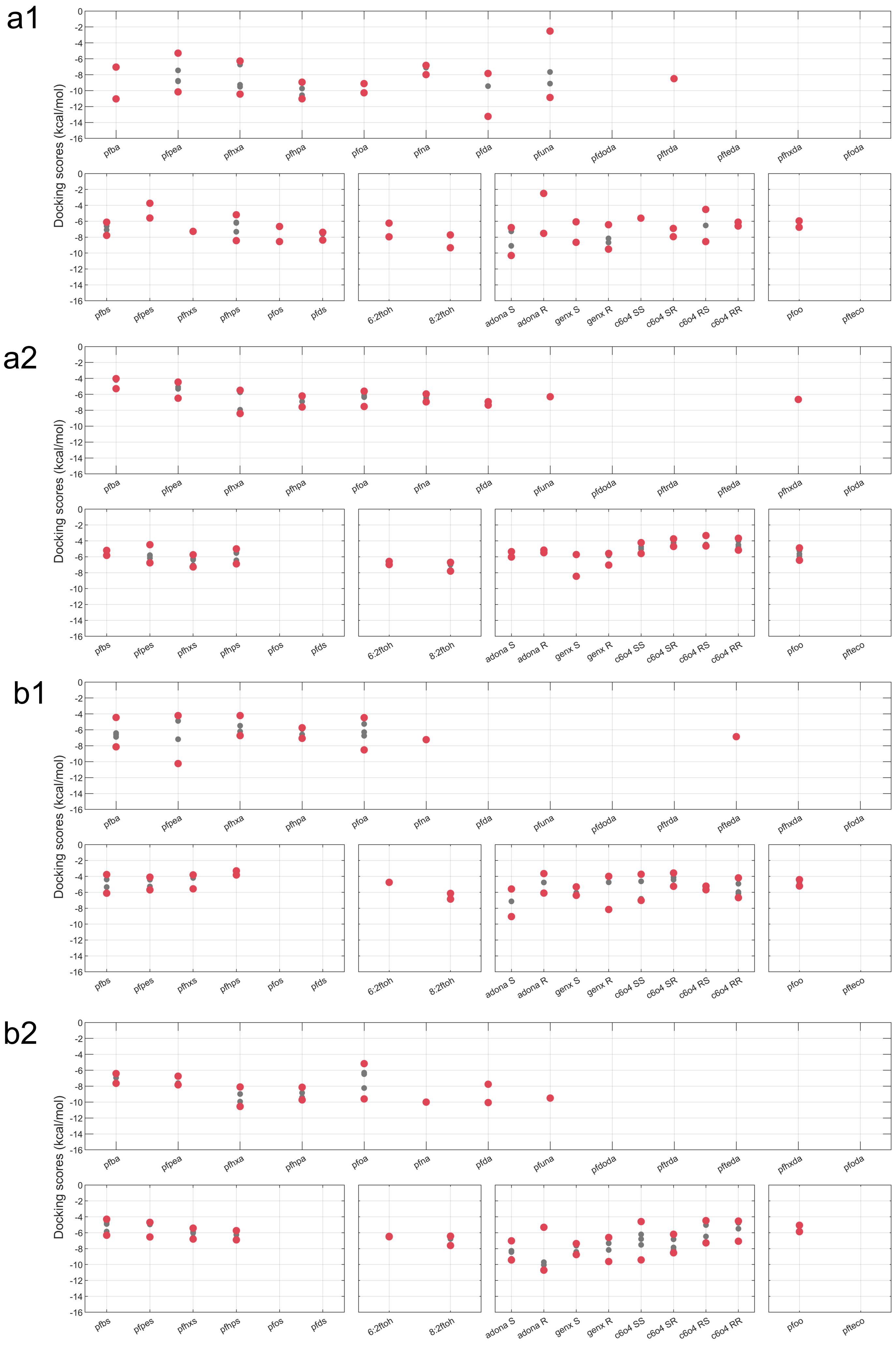
a1
a2
b1
b2
Docking scores (kcal/mol)
0
-2
-4
-6
-8
-10
-12
-14
-16
pfba
pfpea
pfhxa
pfhpa
pfoa
pfna
pfda
pfuna
pfdoda
pftrda
pfteda
pfhxda
pfoda
pfbs
pfpes
pfhxs
pfhps
pfos
pfds
6:2ftoh
8:2ftoh
adona S
adona R
genx S
genx R
c6o4 SS
c6o4 SR
c6o4 RS
c6o4 RR
pfoo
pfteco

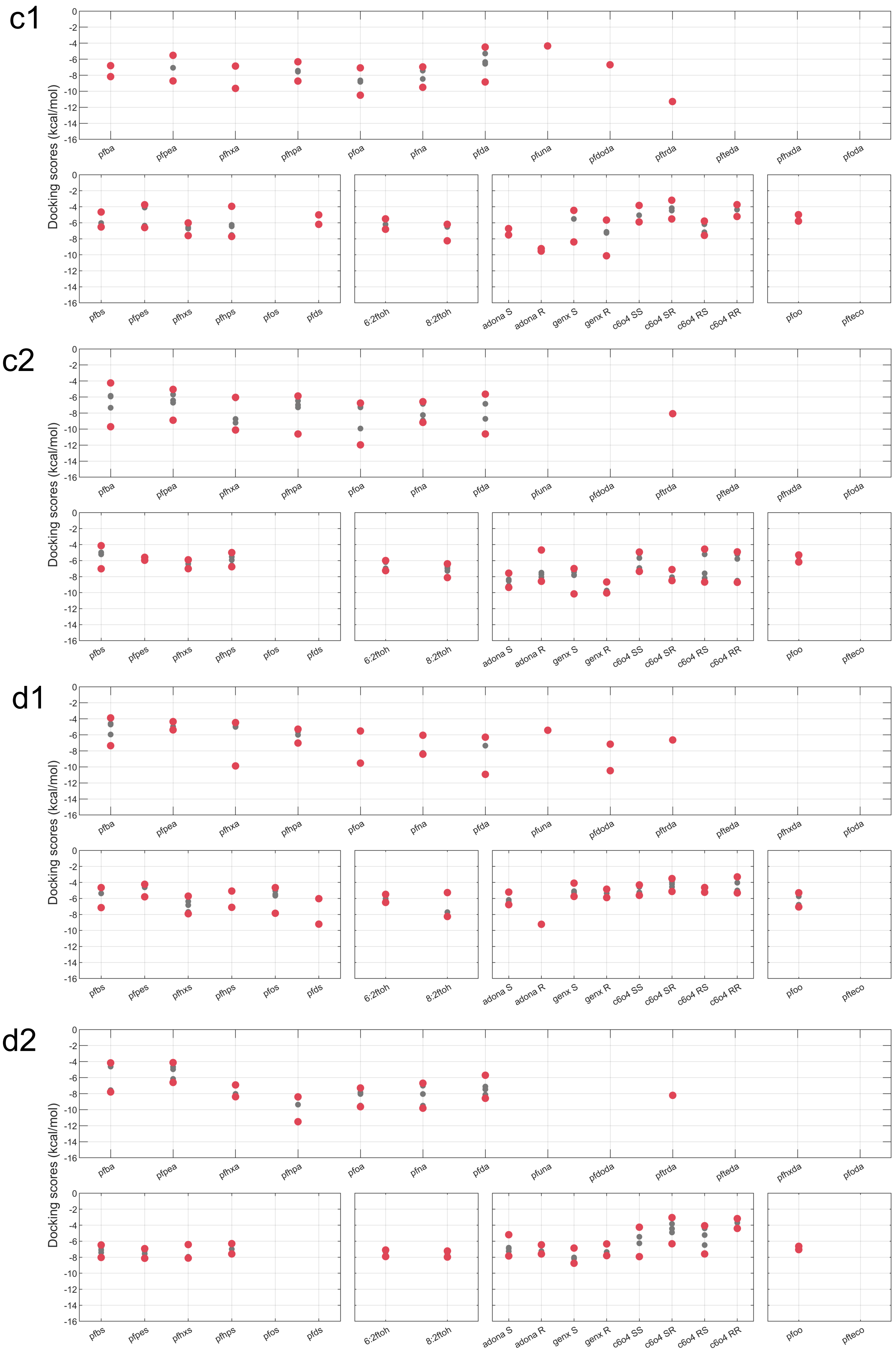

Figure S21. Docking scores (kcal/mol) for PFAS series docked in OATs and URAT1. **a1.** OAT1 outward; **a2.** OAT1 inward; **b1.** OAT3 outward; **b2.** OAT3 inward; **c1.** OAT4 outward; **c2.** OAT4 inward; **d1.** URAT1 outward; **d2.** URAT1 inward.

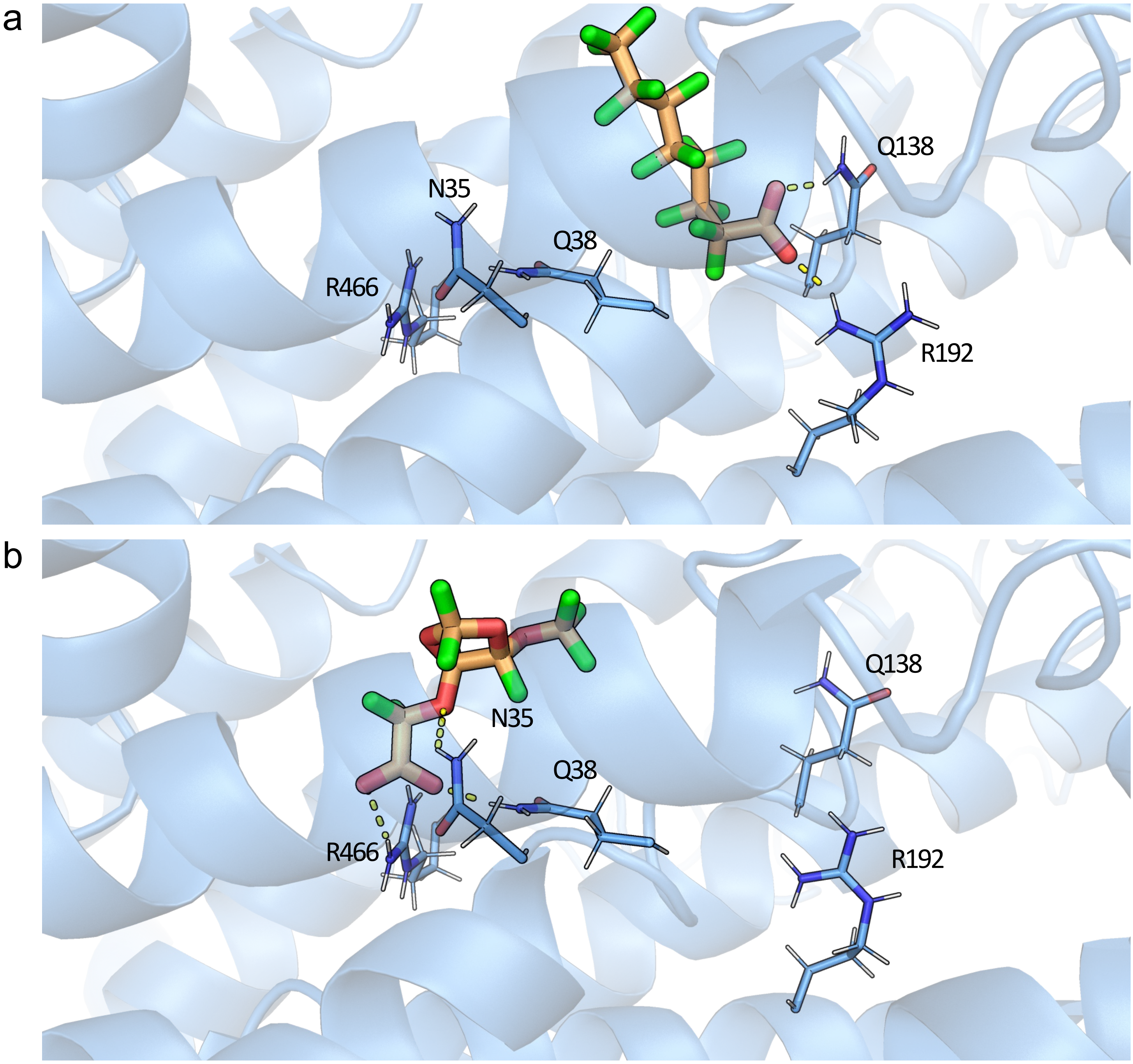

Figure S22. Visual representation of the docking poses of PFOA (**a**) and cC6O4 (**b**) in OAT1 outward. The highest affinity poses were selected for visualization. The protein is shows as cartoon, the ligands and crucial residues in capped sticks, H-bonds as yellow dashed lines. The exterior of the cell, in OAT1's case the lumen side of the kidney cell, is on the right of the figures, while the interior of the cell is on the left side.

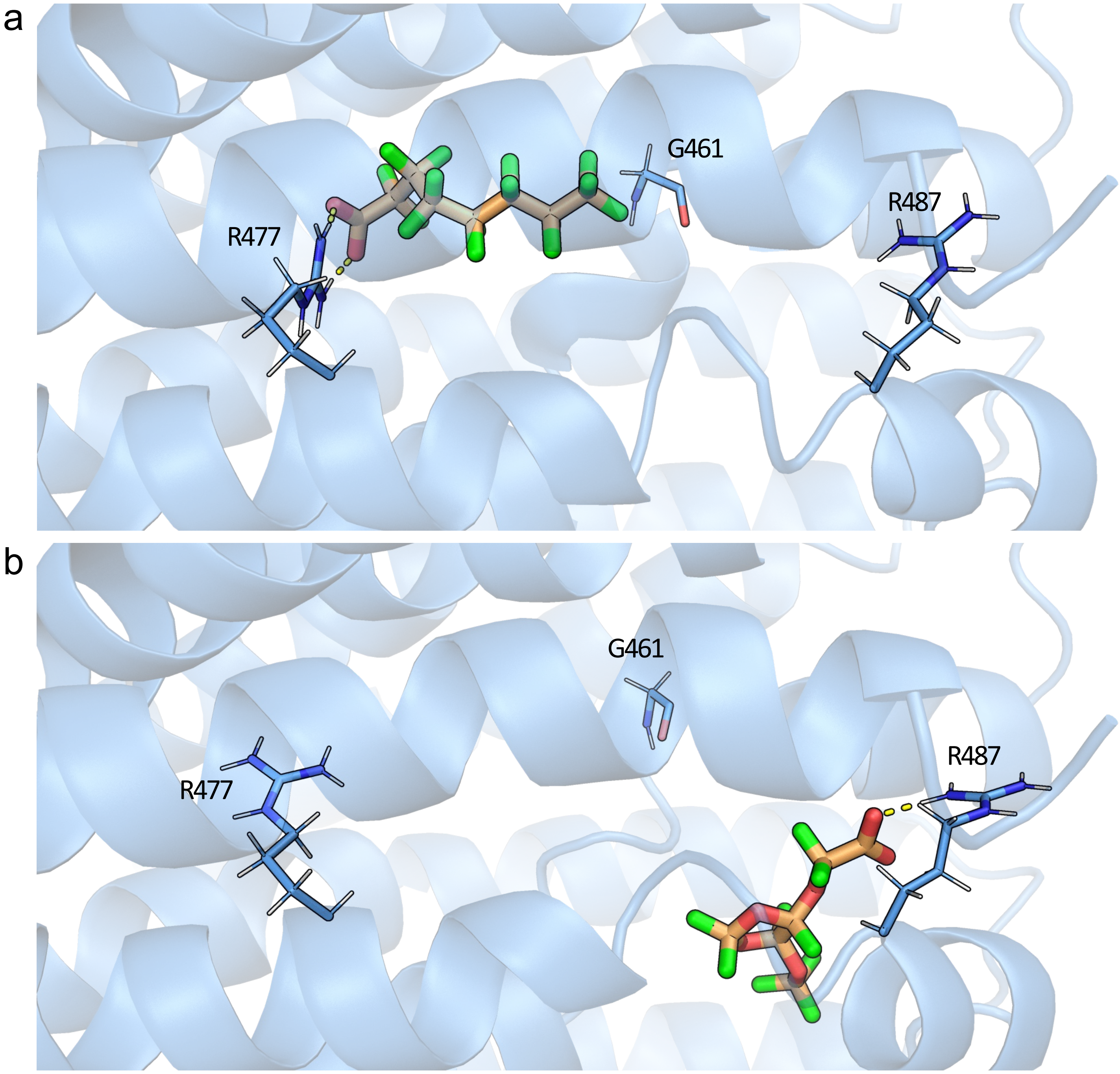

Figure S23. Visual representation of the docking poses of PFOA (**a**) and cC6O4 (**b**) in URAT1 outward. The highest affinity poses were selected for visualization. The protein is shows as cartoon, the ligands and crucial residues in capped sticks, H-bonds as yellow dashed lines. The exterior of the cell, in OAT1's case the lumen side of the kidney cell, is on the right of the figures, while the interior of the cell is on the left side.
